# Using phidelta diagrams to discover relevant patterns in multilayer perceptrons

**DOI:** 10.1038/s41598-020-76517-0

**Published:** 2020-12-07

**Authors:** Giuliano Armano

**Affiliations:** Department of Mathematics and Computer Science, University of Cagliari, Cagliari, Italy

**Keywords:** Computer science, Information technology

## Abstract

Understanding the inner behaviour of multilayer perceptrons during and after training is a goal of paramount importance for many researchers worldwide. This article experimentally shows that relevant patterns emerge upon training, which are typically related to the underlying problem difficulty. The occurrence of these patterns is highlighted by means of $\langle \varphi ,\delta \rangle$ diagrams, a 2D graphical tool originally devised to support the work of researchers on classifier performance evaluation and on feature assessment. The underlying assumption being that multilayer perceptrons are powerful engines for feature encoding, hidden layers have been inspected as they were in fact hosting new input features. Interestingly, there are problems that appear difficult if dealt with using a single hidden layer, whereas they turn out to be easier upon the addition of further layers. The experimental findings reported in this article give further support to the standpoint according to which implementing neural architectures with multiple layers may help to boost their generalisation ability. A generic training strategy inspired by some relevant recommendations of deep learning has also been devised. A basic implementation of this strategy has been thoroughly used during the experiments aimed at identifying relevant patterns inside multilayer perceptrons. Further experiments performed in a comparative setting have shown that it could be adopted as viable alternative to the classical backpropagation algorithm.

## Introduction

Artificial Neural Networks (ANNs), and in particular multilayer perceptrons (MLPs), are a tool of paramount importance for machine learning and pattern recognition, thanks to their effectiveness and flexibility. As the classical formulation of the backpropagation algorithm (see for example Rumelhart et al.^1^) follows the guidelines of gradient descent, the mainstream in the research on ANNs has been focusing on how to escape from local minima (see for example Lo et al.^2^ and Atakulreka and Sutivong^3^). Related works explore the hypothesis that local minima lead to performances that are similar to the one obtained by reaching the global minimum (see for example Choromanska et al.^4^). The problem of saddle points has also been investigated, trying to verify the assumption that finding local minima is actually not a problem (see for instance Pascanu et al.^5^ and Dauphin et al.^6^ on this matter). Further studies have been performed by relaxing the classical training strategy in various ways. Let us recall only the proposals that fall under the umbrella of stochastic gradient descent (SGD), as they have become a de facto standard for training modern MLP architectures. These proposals share a common ancestor, i.e., the pioneering work of Robbins and Monro^7^. Recent variations of this motif include in particular Adagrad^8^ RMSProp (by Tieleman and Hinton, 2012—unpublished), Momentum^9^ Adam^10^ and kSGD^11^. Without claiming to be exhaustive, other proposals made in recent years that go beyond the cited SGD strategy are briefly recalled hereinafter. Lee et al.^12^ explore a novel approach to credit assignment in deep neural networks (DNNs), called target propagation. The underlying idea is to compute, at each layer, targets rather than gradients. Like gradients, targets are propagated backwards; however, this process relies on autoencoders (see Hinton and Salakhutdinov^13^). Lillicrap et al.^14^ propose a mechanism, called feedback alignment, which assigns blame by multiplying errors even by random synaptic weights. The authors show that this mechanism can transmit teaching signals across multiple layers of neurons and performs as effectively as backpropagation on a variety of tasks. Following the work on feedback alignment, Nøkland^15^ shows that the error can also be propagated, through fixed random connections, directly from the output layer to each hidden layer. Moreover, Frerix et al.^16^ propose an algorithm that takes implicit rather than explicit gradient steps to update the network parameters during training.

With the advent of DNNs, a renewed interest has been shown in the problem of how to get insights about the inner behaviour of neural architectures. Alain and Bengio^17^ propose to measure how suitable are for classification the features that occur at every layer of a model. To this end, the authors use linear classifiers (named probes), which are trained independently of the model itself. Information theory has also been used as prominent formal tool in the attempt of unveiling relevant properties of ANNs equipped with multiple layers. According to this grounded view, entropy and related concepts (in particular, mutual information) have been used to capture the flow of the information throughout the layers of neural architectures. Achille and Soatto^18^ show that invariance to nuisance factors in a DNN is equivalent to information minimality of the learned representation, and that stacking layers and injecting noise during training naturally bias the network towards learning invariant representations. Following the work of Tishby et. al^19^ and of Tishby and Zaslavsky^20^, Shwartz-Ziv and Tishby^21^ point out that any internal representation of a DNN can be seen as the combination of an encoder and a decoder. Starting from this concept, the authors show that this combination can be quantified by its information plane coordinates and that the optimal representations are constrained by the Information Bottleneck bound. Moreover, they show that most of the training epochs in standard deep learning are spent on input compression rather than on fitting the training labels. The authors emphasize that this generalization mechanism is unique to DNNs and absent in classical networks equipped with one hidden layer. Moreover, they show that the training time is dramatically reduced when adding more hidden layers. Wickstrøm et al.^22^ suggest that the goal of a network is to optimize, for each layer, the trade-off between compression and prediction. They propose a framework for information plane analysis able to shed new light on small-scale DNNs, with the final goal of analysing contemporary large-scale DNNs.

According to Yang et al.^23^, performance and model explainability are the two most important objectives when developing machine learning algorithms to solve real-world problems. More recently, Yu and Principe^24^ point out that, despite their great success in practical applications, there is still room for analysing DNNs from a theoretical (and systematic) perspective. These concerns also originated a DARPA program launched in 2016, aimed at improving eXplainability in AI systems^25^. The goal of this program is to enact the definition or modification of machine learning techniques aimed at producing explainable models. Relevant issues along this perspective are (i) the types of measures to be adopted, being aware that accuracy, in its standard definition, may not be a proper tool for revealing the quality of a learned model; and (ii) the need for suitable tools aimed at investigating and visually inspecting the inner behaviour of MLPs. In a 2010 article, Valverde-Albacete and Peláez-Moreno^26^ propose the adoption of entropic measures based on the confusion matrices that characterize the behaviour of multi-class classifiers. The authors end up with a measure reported in a three-dimensional entropy space, which can be further projected in a two-dimensional space as De Finetti entropy diagram. In a subsequent article^27^ the same authors point out that the most widely acknowledged measure of performance, i.e., accuracy, may fail to capture crucial information transfer in the classification task. To show evidence of this unwanted behaviour, the authors use the entropy triangle to perform a combinatorial analysis on variously sized confusion matrices. In fact, it is well known that accuracy can give information about the actual discrimination capability of a classifier only when negative and positive data are perfectly balanced. To deal with the difficulties of applying accuracy in its classical definition, the authors propose another measure, called “entropy-modulated accuracy”, in which the influence of the input distribution is factored out. According to the author claims, this is a more natural measure of classification performance than accuracy when the heuristic to maximize is the transfer of information through the classifier instead of classification error count. In 2015, Armano^28^ proposed $\langle \varphi ,\delta \rangle$ measures and diagrams, the latter being a diamond-shaped 2D graphical tool devised to support the work of researchers on binary classifier performance evaluation and on feature assessment. This proposal is rooted on specificity and sensitivity, as done for ROC curves^29^. Hence, the visual inspection proceeds by pretending that the underlying data are in fact balanced. When used for feature assessment, these diagrams allow to depict the so-called class signature (or *signature*, for short), which is obtained by reporting the behaviour of each feature, seen as an elementary classifier, with respect to the positive category.

Being aimed at at shedding new light on the inner behaviour of MLPs, this article falls in the the frame of eXplainable AI. The underlying conjecture is that specific patterns are expected to arise at the hidden layers of an MLP upon training. In turn, this conjecture is supported by the assumption that MLPs are very effective feature encoders. Not incidentally, this assumption is in full accordance with the cited analysis of Swhartz-Ziv and Tishby^21^, who observe that—fixing any hidden layer—the part of the MLP that originates from the inputs plays the role of encoder, whereas the part headed to the output(s) plays the role of decoder. According to this view, the neurons of a hidden layer can *always* be seen as an alternative representation of the given inputs, as they are in fact projected onto another feature space. Beyond the close connection between this interpretation and entropy-based measures (in particular, mutual information), the assumption is apparently sound also thinking of the MLP as a device that progressively tries to make its embedded features, represented by the neurons of the hidden layers, covariant with one of the given categories. Given that the hidden layers of any MLP can be seen as alternative input sources, they have been investigated by means of $\langle \varphi ,\delta \rangle$ diagrams, with the goal of highlighting the occurrence of relevant patterns able to give information about the generalisation ability of the MLP over the problems at hand. To better investigate the occurrence of these patterns, a training strategy inspired by some relevant recommendations of deep learning (see in particular Bengio et al.^30^, Nøkland^15^ and Juefei-Xu et al.^31^) has also been devised and implemented. In this strategy (called *progressive training*) each layer is trained in isolation, starting with the one in charge of processing the given inputs and going forward until the output layer is reached.

After this clarification, the reader should be aware that this proposal is mainly framed around a *methodological perspective*, aimed at showing that relevant patterns can be found at the hidden layers of an MLP upon training. The remainder of this article is organised as follows: starting with a brief and informal introduction to $\langle \varphi ,\delta \rangle$ diagrams, “Results” reports experimental results, pointing in particular to the rise of relevant patterns inside MLPs able to account for the generalisation process. This section also reports information about the effectiveness of the proposed progressive training algorithm (PT for short, hereinafter), in comparison with the classical backpropagation algorithm (BP for short, hereinafter). “Discussion” makes further comments on the ability of MLPs to act as feature encoders and on the occurrence of relevant patterns inside MLPs. The role of $\langle \varphi ,\delta \rangle$ diagrams in the task of devising more effective training strategies and the possibility of using MLPs and $\langle \varphi ,\delta \rangle$ diagrams in combination to perform multivariate analysis are also discussed. “Methods” illustrates the main characteristics of PT, also focusing on its pragmatic and theoretical roots. “Conclusion” draws conclusions and outlines future work.

## Results

The way the generalisation process takes place in MLPs has been by far the most important aspect that guided the definition of experimental benchmarks. In particular, experiments have been focusing on the rise of success or failure patterns inside MLPs equipped with one or more hidden layers and trained in accordance with BP or PT. This section is organized as follows: after an informal introduction to $\langle \varphi ,\delta \rangle$ diagrams, (i) the occurrence of patterns able to account for success and failure is shown; (ii) the occurrence of failure patterns due to wrong choices on parameter values is highlighted; and (iii) the policy of embedding multiple layers inside an MLP is discussed. As PT has shown to be very effective, some experimental results are also reported at the end of the section.

### Informal introduction to $\langle \varphi ,\delta \rangle$ diagrams

Although in this article $\langle \varphi ,\delta \rangle$ diagrams play a gregarious role, their main concepts are summarized hereinafter for the sake of clarity. Further details on this matter are reported in the Supplementary Information (Supplementary section S1) available online. The interested reader may also find an extensive study on their semantics in Armano and Giuliani^32^.

**Figure 1:**
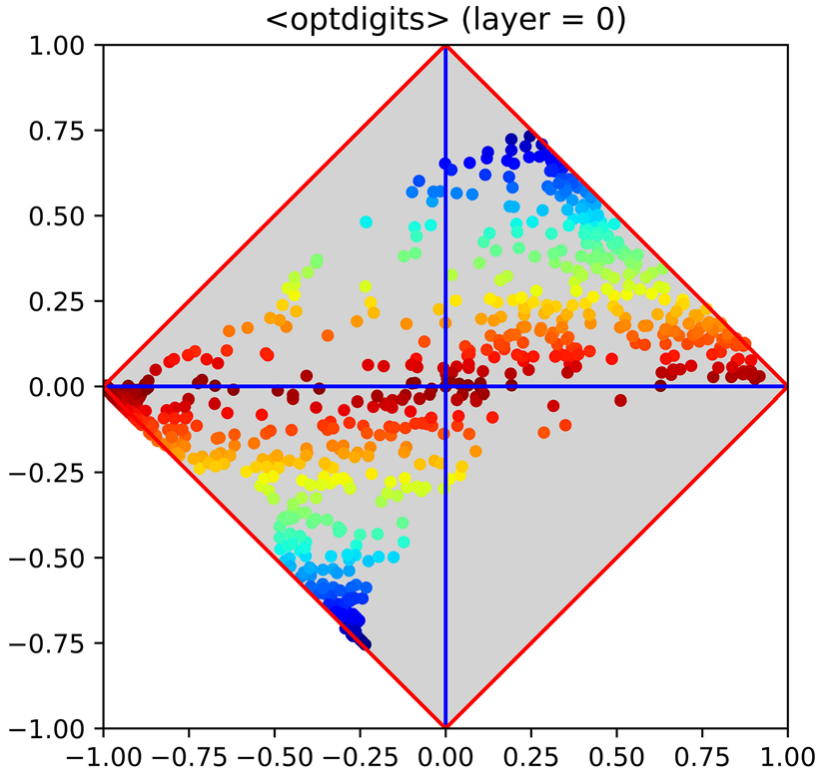
Class signature of the dataset optdigits, downloaded from the UC Irvine machine learning repository (UCI, hereinafter). Each sample is encoded with an image of $32 \times 32$ B/W pixels, for a total of 1024 binary features. The multiclass problem has been binarized considering the digit 0 as positive category and $1, 2, \ldots , 9$ as negative category. Each point in the diagram represents the “performance” of a feature, considered as an elementary classifier. Feature importance is highlighted by a scale of colours: from red (not relevant) to blue (highly relevant). Intermediate values are represented with yellow, green and light blue, depending on the corresponding feature importance (from lower to higher). Due to the presence of several points with high value of |δ|, the problem is expected to be easy.

The measures that give rise to the $\langle \varphi ,\delta \rangle$ space are defined as: $\varphi = \rho - \overline{\rho }$ and $\delta = \rho + \overline{\rho }- 1$, where $\overline{\rho }$ and $\rho$ denote specificity and sensitivity. Upon normalisation, the values of any feature that occurs in the given dataset --as found in the available training samples--can always be seen as the outputs of an elementary (single-feature) classifier. Hence, to draw the class signature of the dataset, first the "performance" of each feature with respect to the positive category is evaluated, and then all corresponding values (in terms of φ and δ) are reported in a diagram. Figure 1 shows an example of how $\langle \varphi ,\delta \rangle$ diagrams can be used for feature assessment, by depicting the signature of the dataset *optdigits*. It is worth to highlight that φ coincides with the horizontal axis, whereas δ with the vertical one. In the cited figure, points have different colours, which account for their importance; in particular, blue points are assumed to be highly useful for the classification process and vice versa for red points. However, note that hereinafter the colour map will be automatically rescaled according to the difference between minimum and maximum value of |δ|. This choice has been taken to facilitate the reader at visually ranking points even when the values of |δ| have low dynamics. According to the given definitions, both measures range in the interval [− 1, + 1], and their underlying semantics is the following: $\varphi$ estimates the *bias* of a feature with respect to the positive (and negative) category on the given dataset, whereas (though stretched in $[-1,+1]$) $\delta$ gives the *accuracy* of a feature with respect to the positive category. Note that, being given in terms of specificity and sensitivity, φ and δ are both independent of the actual balancing between negative and positive samples. It can be easily shown that $\varphi$ is the locus of points such that the mutual information between a feature and the positive or negative category drops to zero. Hence, features laying close to this axis (i.e., such that $\vert \delta \vert \approx 0$) are expected to provide limited or negligible support to the classification process. This is true for the whole axis, although with different semantics (in particular, features independent of either class label lay at the crossing of the $\varphi$ and $\delta$ axes). As for $\delta$, by construction, it gives information about the degree of agreement (or disagreement) between a feature and the positive category. In case of agreement (upper corner), the feature is said to be covariant with the positive category, whereas in case of disagreement (lower corner) it is said to be contravariant. Features whose $\delta$ value is close to the upper or lower corner are expected to give strong support to the classification process. The fact that both highly covariant and highly contravariant features are equally important should not be surprising, as selecting a class as positive or negative is just an arbitrary choice.

### Patterns that arise on easy and difficult problems

**Figure 2:**
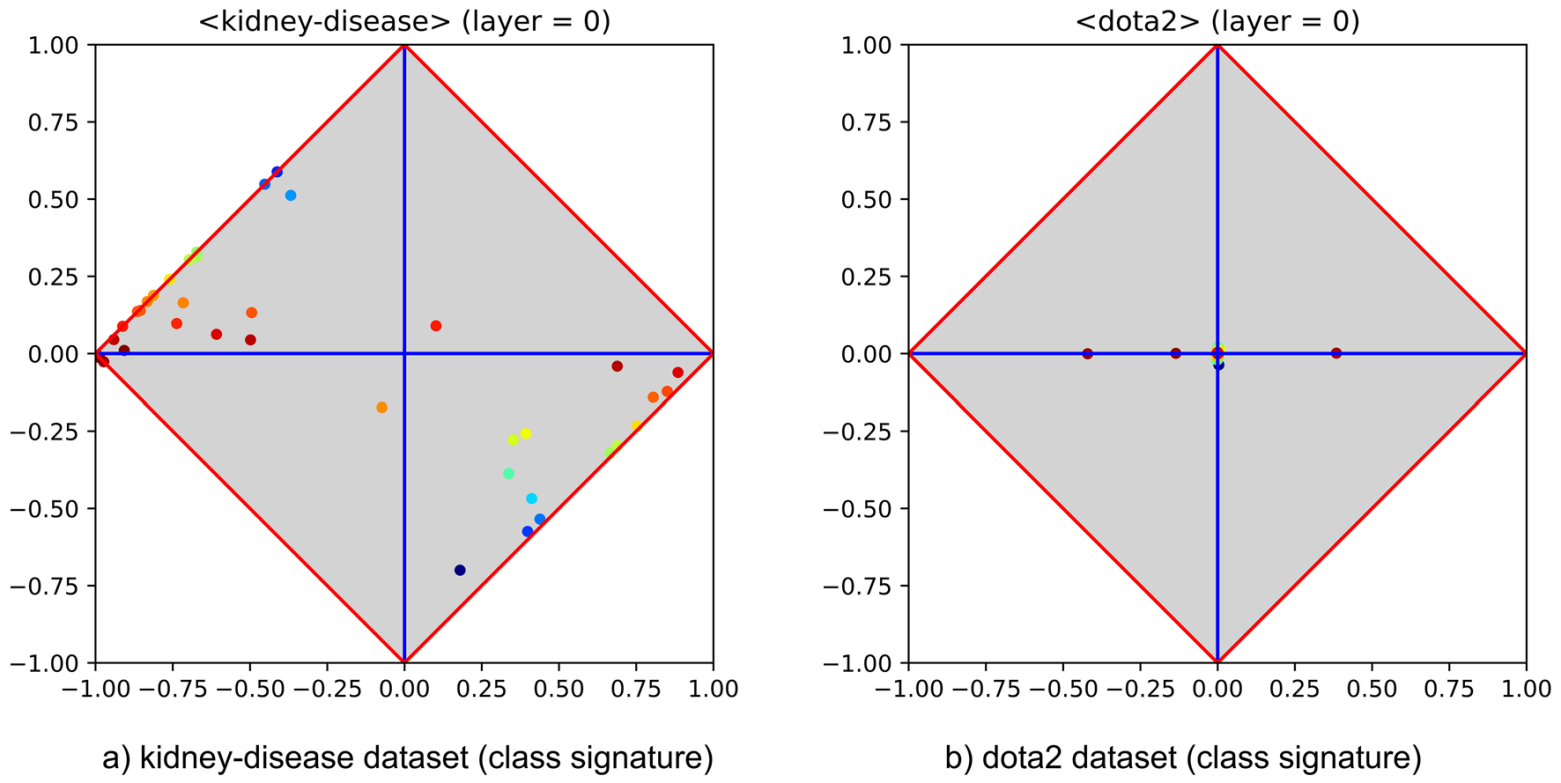
Two typical class signatures, for easy and difficult classification problems. In particular, the toy problem kidney-disease is reported at the left-hand side, whereas the (expected to be) difficult problem dota2 is reported at the right-hand side. Both datasets have been downloaded from UCI.

The first experimental benchmark has been set with the goal of identifying whether specific patterns occur inside MLPs, able to account for their generalisation ability over the problem at hand. To this end, both easy and difficult problems have been investigated. Figure 2 reports the signatures of two exemplar problems, i.e., kidney-disease and dota2. The left-hand side points out that several features of kidney-disease are in high agreement with the negative category. Having *at least one* feature highly covariant with either category (i.e., with high $\vert \delta \vert$) is a sufficient condition that guarantees the easiness of a problem, due to the expected performance of the corresponding elementary classifier on the test set (in fact, the blue feature not far from the bottom would allow to reach an accuracy of about $85\%$). Conversely, the right-hand side shows that in dota2 a very poor agreement holds between features and categories, as all features therein lay on (or are very close to) the $\varphi$ axis. Notably, having *all* features with low $\vert \delta \vert$ is not a necessary condition for the problem to be difficult, as by construction a class signature is able to report only the agreement/disagreement between each feature taken in isolation and the positive category. However, using their capability of acting as feature encoders, MLPs can be used to put into evidence if any useful combination of input features holds.

**Figure 3:**
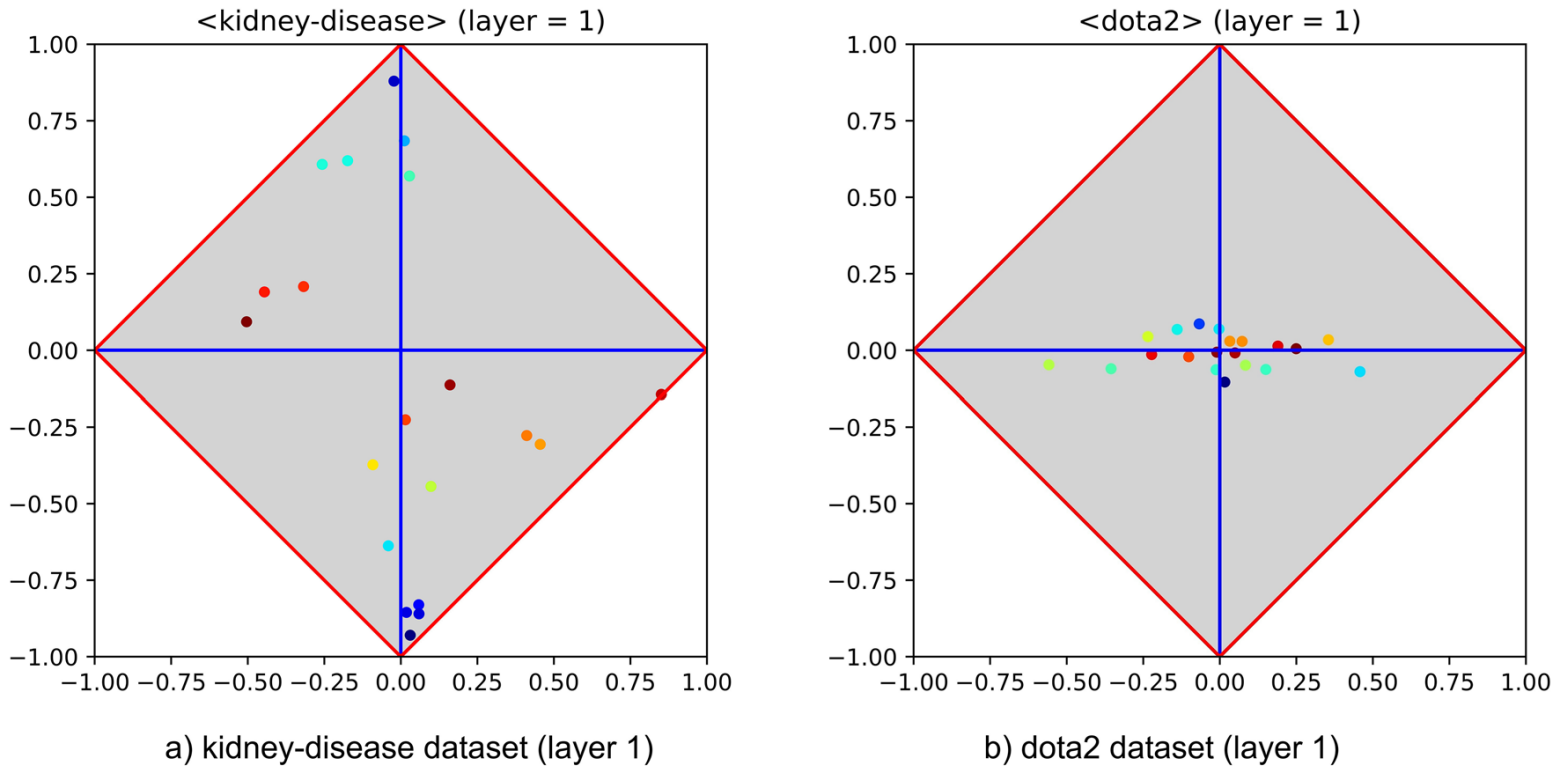
Hidden layer class signatures of MLPs trained on kidney-disease and on dota2 (left- and right-hand side). The diagrams highlight that the neuron outputs obey to very different patterns (i.e., success and failure, respectively).

To verify that the first problem is in fact easy and to assess whether the second is difficult or not, an MLP with a single hidden layer and equipped with 20 neurons has been trained on both problems using 10-fold cross validation. Learning rate, momentum and number of epochs have been set to typical values (i.e., 0.01, 0.05 and 40, respectively), whereas sigmoid has been used as activation function. The easiness of the former problem has been confirmed by $100\%$ of accuracy, and the difficulty of the latter has been confirmed by an accuracy of about $57\%$, which is very close to random guessing. More interestingly, the hidden layers found after training the MLP on the selected problems are very different and deserve attention. Let us separately examine the $\langle \varphi ,\delta \rangle$ diagrams drawn by considering the outputs of each hidden layer as they were in fact *input features*. The left-hand side of Fig. 3, which reports the hidden layer of the MLP trained on the dataset kidney-disease, highlights the occurrence of a characteristic pattern. In particular, part of the neuron outputs lay close to the upper corner while most of the others lay close to the lower corner. The interpretation of this feature recombination process is clear: the MLP has been able to generate new features highly covariant (upper corner) or highly contravariant (lower corner) with the positive category (recall that both covariant and contravariant features are highly discriminant). In the event that the training set is representative of the dataset at hand, finding this pattern implies that the MLP has been able to come up with a very good predictive model through generalisation. The right-hand side of Fig. 3, which reports the hidden layer of the MLP trained on the dataset dota2, highlights the occurrence of a completely different pattern. In particular, all neurons therein lay close to the φ axis, several of them being also close to the centre of the diagram. Yet, the interpretation of this feature recombination process is clear: the MLP has not been able to generate any new feature highly covariant or contravariant with the positive category. Rather, such hidden layer highlights the inability to provide a generalisation by resorting to random guessing, meaning that those neurons and the positive or negative category tend to be statistically independent.

### Failure patterns that may occur depending on training parameters

**Figure 4:**
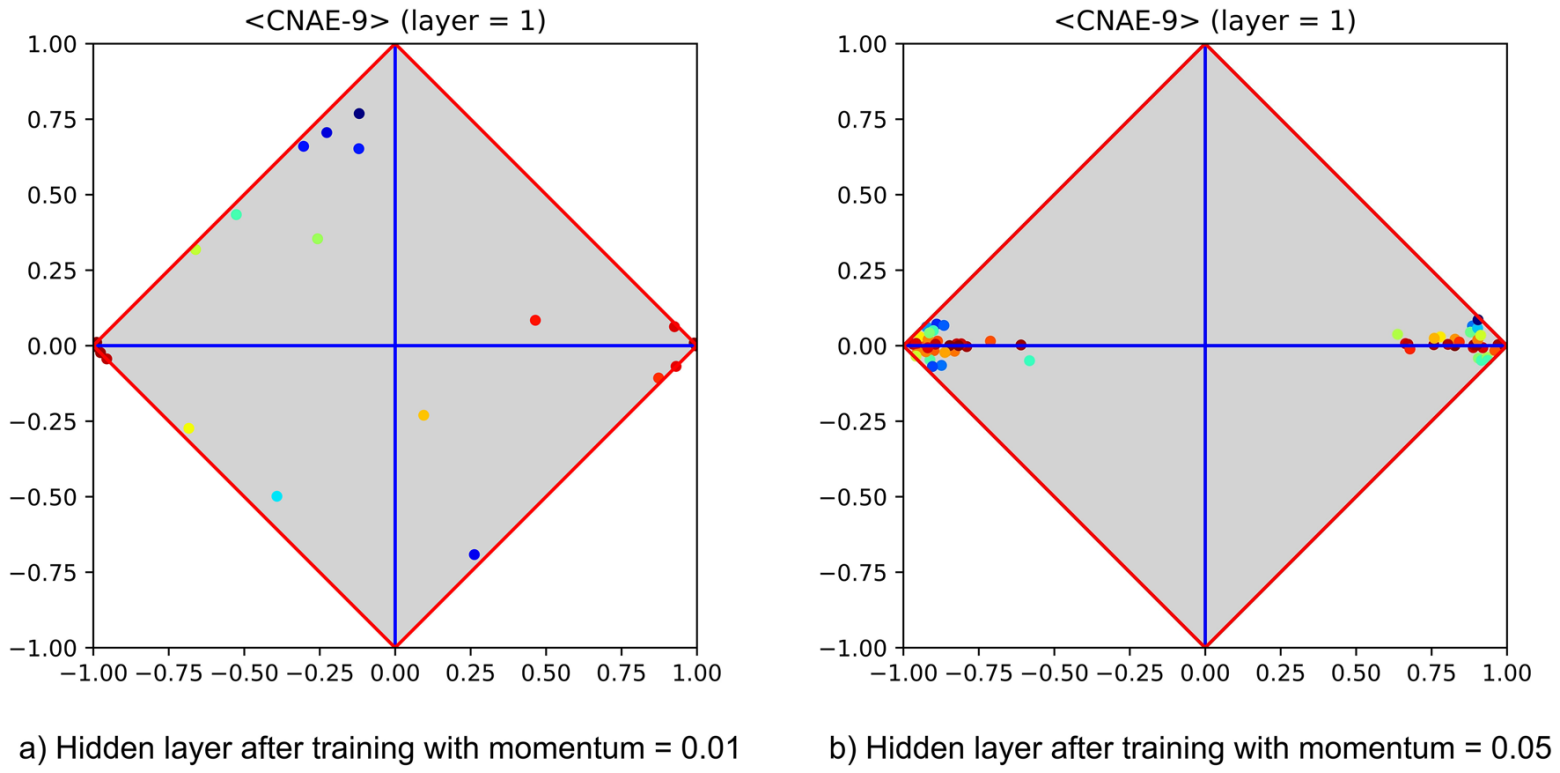
Depicting the hidden layer of an MLP trained with different values of momentum on the CNAE-9 dataset (from UCI). In particular, the hidden layer at the left-hand side shows that the MLP has been able to generalise, despite the fact that some neurons (i.e., those located at the left- and right-hand corners) operate in saturation, whereas the other highlights a clear pattern of failure, as all neurons operate in saturation.

Understanding the impact of MLP parameters on the generalisation ability of the learning algorithm is a main issue. In particular, it is well known that changes on learning rate and momentum must be dealt with care, otherwise—also depending on the selected activation function—the learning process might end up with a failure. Just as an example, let us consider how the generalisation ability of an MLP equipped with one hidden layer depends on different values of momentum. Figure 4 reports the hidden layers obtained after training the dataset CNAE-9 with two different values of momentum. The left-hand side shows the signature obtained with momentum equal to 0.01 and the right-hand side the one obtained with momentum equal to 0.05. The former case highlights a pattern of generalisation success, whereas the latter highlights a clear pattern of generalisation failure. Note that in both layers there are neurons whose $\langle \varphi ,\delta \rangle$ values that do not show any useful correlation with the positive or negative category (see the left- and the right-hand corners of the diagrams). Indeed, any such neuron would clearly emit $-1$ (left) or $+1$ (right), regardless of the actual input. This phenomenon occurs on neurons that operate in saturation, and—in absence of further neurons with medium/high covariance or contravariance with the positive category—it implies a generalisation failure. It is worth noting that, together with a more careful choice of training parameters, the occurrence of residual neurons that underwent saturation can be dealt with in several ways. For instance, alternative activation functions could be adopted, e.g., rectified linear unit (ReLU, for short)—see, for example, Hahnloser et al.^33^ and Glorot et al.^34^. In the event that a layer-wise training strategy is adopted, the best way for dealing with this problem would be to prune these residual neurons off, for they cannot carry any useful information onwards.

### Improving the generalisation ability of MLPs by adding layers

Although it is widely acknowledged that an MLP with a single hidden layer can be a universal function approximator^35,36^ with the rise of deep learning it has been shown that many real-world problems allow very compact representations with increasing number of layers—see, in particular, Bengio^37^. According to this view, one can hypothesize that deploying a difficult problem over an MLP with more than one hidden layer may help its generalisation ability. A simple way to verify this hypothesis consists of thrusting each intermediate layer, but the last, to act as feature encoder. Unfortunately, classical backpropagation is not compliant with this perspective, as in principle all layers are concurrently updated every time a training sample is processed. Being aware of this limitation, PT has been used in place of BP, the main characteristics of the former being its ability of training each hidden layer in isolation, starting from the first and going onwards. Technical details on this layer-wise strategy are given in “Methods” section.

**Figure 5:**
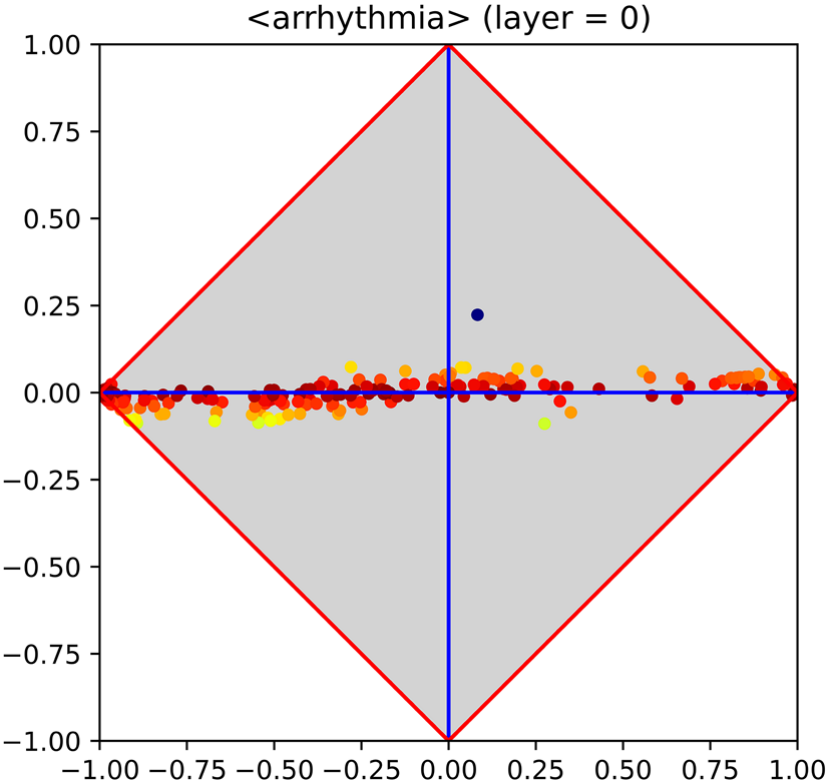
Class signature of the arrhythmia dataset (from UCI). The problem is expected to be difficult, as almost all features lay in proximity of the $\varphi$ axis; the only exception being the blue point at the upper-right part of the diagram, which shows a small, but not negligible, correlation with the occurrence of arrhythmia. Besides, that point corresponds to the binary feature *sex*={M,F}, which is in accordance with the existing statistics about this disease.

**Figure 6:**
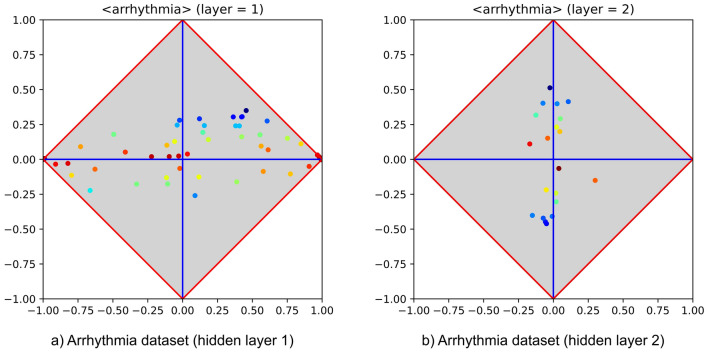
Inner behaviour of an MLP, trained with PT, on the arrhythmia dataset (from UCI). The two hidden layers (with 50 and 20 hidden units, respectively) are entrusted with very different tasks: the one whose class signature is shown at the left-hand side basically performs feature extraction, whereas the other (right-hand side) is responsible for generalisation.

Let us examine the behaviour of an MLP that underwent progressive training using an example in which the problem at hand is apparently difficult. Figure 5 reports the signature of the classical dataset arrhythmia. This signature suggests that the problem is expected to be difficult, as almost all features lay in proximity of the $\varphi$ axis. Running 10-fold cross validation on an MLP that embeds a single hidden layer allowed to reach an accuracy of about $65\%$, regardless of the adopted MLP parameters and of the number of hidden neurons (i.e., 10, 20 and 50 at different trials). On the contrary, training an MLP that embeds two hidden layers using PT allowed to obtain an accuracy of about $75\%$. Figure 6 shows the signature corresponding to each hidden layer, making clear that they have separate responsibilities. In particular, the first performs a successful feature encoding, so that several new “features” are now more correlated with the positive or negative category. The second layer highlights that the attempt of finding a good model (upon the outputs of the previous layer) has been almost successful, as some neurons are now covariant with the positive category, whereas others are contravariant. It is clear, however, that in fact the success pattern found for the arrhythmia dataset lies in between the patterns of failure and success. To what extent any such pattern approximates failure or success depends on the distance of the neural clusters from their reference (upper or lower) corner. This phenomenon may be due to the residual noise or to the limits of classical gradient descent. In either case, this “half-way” pattern deserves attention, being highly relevant in various aspects concerning the behaviour of MLPs.

### Effectiveness of progressive training

Beyond the need of making clearer the process of feature encoding and pattern formation inside an MLP, PT has also shown to be very effective. Three kinds of experiments are reported hereinafter: (i) a preliminary experiment on a synthetic dataset in which all features have been randomly generated, and two of them determine the labelling of samples throughout the *xor* logical function; (ii) experiments performed on some non-trivial datasets from UCI; and (iii) experiments on well-known medium-size datasets, showing that relevant patterns can be found also in problems characterised by high number of samples and/or features. All experiments shed light on the fact that relevant patterns occur regardless of the size of datasets and regardless of the origin of data (i.e., synthetic or real).

#### Experiments on the *xor* dataset

**Figure 7:**
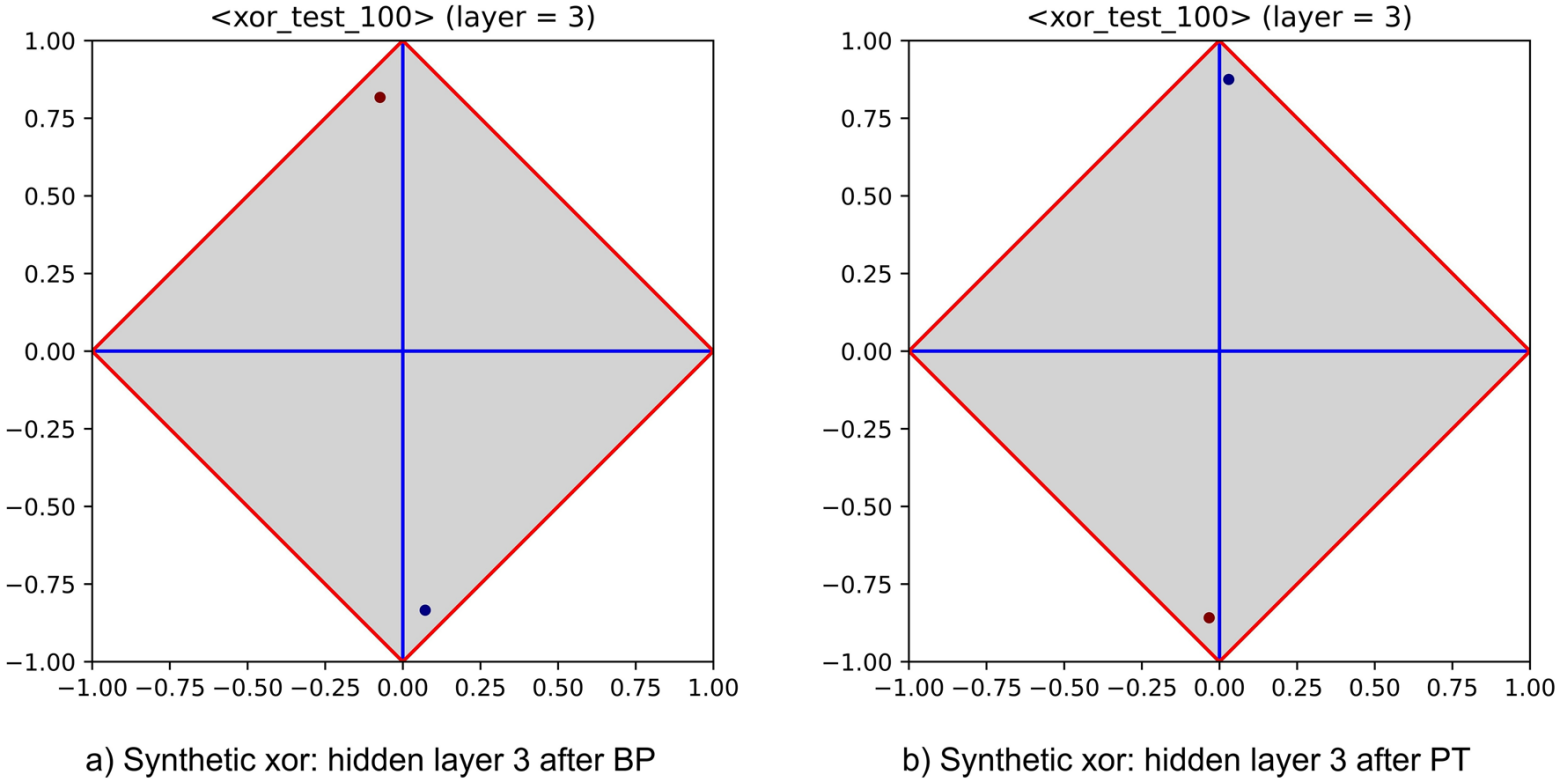
Success patterns of an MLP equipped with three hidden layers (embedding 10, 4 and 2 neurons) trained with BP and PT (left- and right-hand side), on the synthetic xor dataset. Note that the rise of a success pattern is clearer for PT.

**Figure 8:**
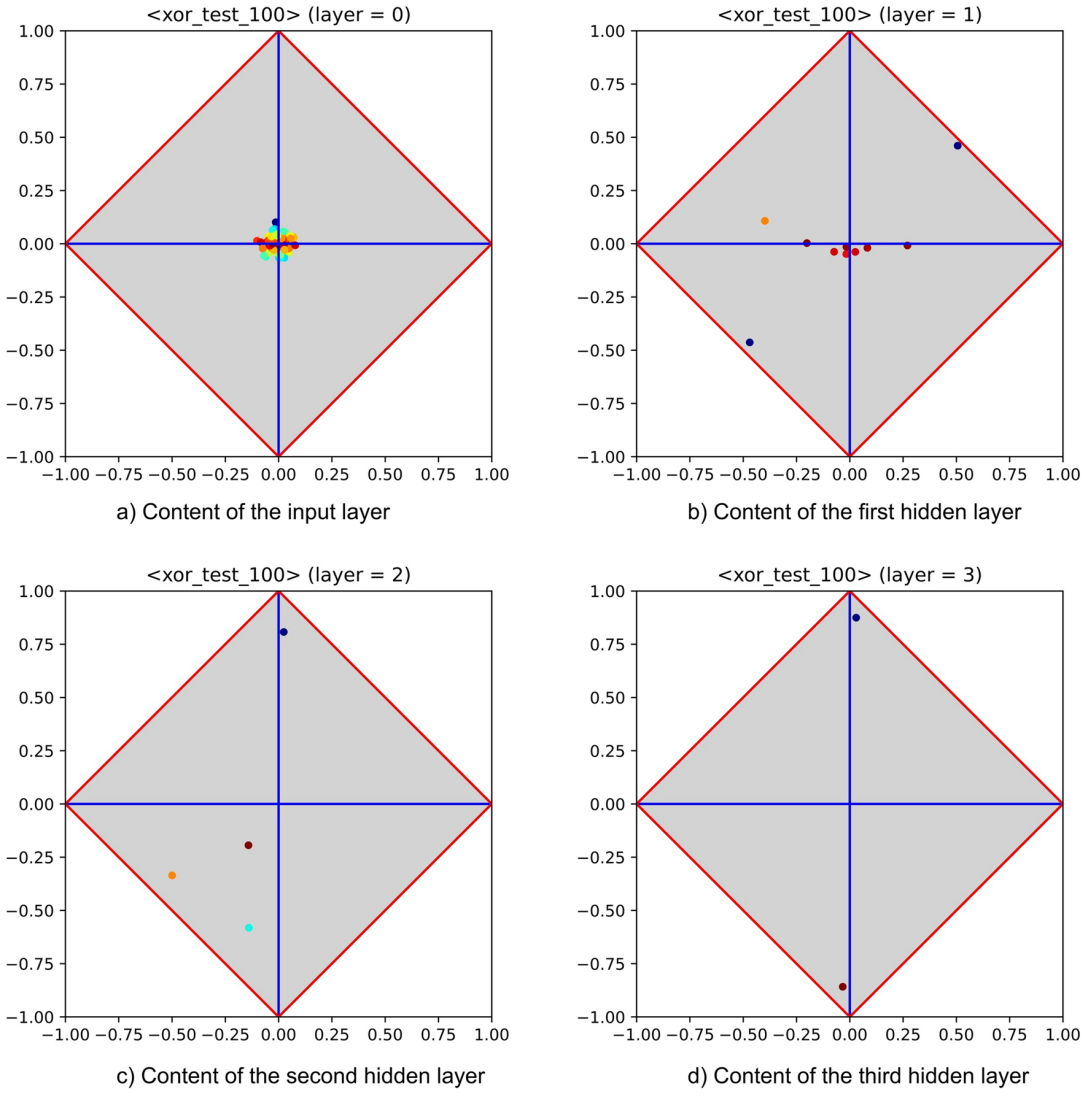
Feature extraction performed by an MLP equipped with three hidden layers (with 10, 4, and 2 neurons) and trained with PT on the synthetic xor dataset. The sequence highlights the role of feature extractor played by the MLP. Note that, by construction, each feature taken in isolation—including those used to generate the labelling—is almost completely independent of the class label (upper left-hand diagram).

As preliminary experiment, the power of layer-wise training has been assessed on a variant of the classical *xor* problem. This groundwork has been devised to verify that the ability of MLPs to combine features persists while moving from BP to PT. The corresponding experimental setup is the following: a synthetic dataset with 98 real-valued features and 1000 samples has been generated. Afterwards, two logical features have been added, randomly selecting—for each sample—their values. Then, the labelling of each sample has been set by calculating the *xor* between the two extra features. An MLP has been trained on this dataset, first using BP and then using PT. The corresponding architecture (i.e., three hidden layers with 10, 4, and 2 neurons) has been left unchanged. To prevent that experiment outcomes were biased by a single run, multiple runs have been performed, with typical values for learning rate and momentum (i.e., 0.01, 0.05, respectively). A negligible classification error (i.e., about $0.5\%$) has been evidenced during experiments, meaning that in both cases the MLP was able to identify the two relevant input features. Figure 7 illustrates the content of the third hidden layer, as obtained by enforcing both kinds of training strategies (these contents have been extracted from a single, though representative, run). The cited figure highlights that both MLPs are able to cope with the *xor* problem. However, the rise of a success pattern is clearer for PT. The feature extraction process is highlighted by Fig. 8, which summarizes the informative content of the input layer and of all hidden layers, as obtained by running PT. Notably, the input layer makes clear that this problem cannot be solved by univariate analysis, as all features—taken in isolation—are almost completely independent of the class label (recall that perfect independence occurs at the crossing between horizontal and vertical axis). Nevertheless, the first hidden layer shows that several combinations of input features are in fact relevant for the classification process. An initial attempt of reaching a success pattern is then shown at the second layer, whereas a full success pattern occurs at the third hidden layer.

#### Experiments on non-trivial datasets from UCI

Some preliminary comments follow on experimental setup. A fixed policy has been adopted for parameter setting, with the goal of ensuring a fair assessment for the algorithms under testing. In particular, learning rate and momentum have been set to 0.01 and 0.05, whereas the number of epochs to 40. To ensure statistical significance, training and test runs have been repeated 100 times for each dataset using random splitting. A two-sample Welch *t*-test has been evaluated on available data. The confidence level for *p*-values has been set to 0.05. As for MLP architectures, a fixed choice has been adopted as well. In particular, a hidden layer equipped with 30 neurons has been used for experiments made on MLPs equipped with a single hidden layer (BP only), whereas a network shape with $\langle 24, 10, 6 \rangle$ neurons has been used for experiments made on MLPs equipped with more hidden layers (for both BP and PT). The amount of variations being sizeable, commands have been run in batch mode. Some details about the format of batch command files are given in the Supplementary Information available online (Supplementary section S2). Also log files concerning experimental results (which include confusion matrix, specificity, sensitivity, accuracy, $\varphi$ and $\delta$) are automatically stored in a similar format during each run; in so doing, any further analysis is greatly simplified.

As for the criteria used for assessment, they have been derived from two typical perspectives: one focused on the classifier behaviour under the hypothesis that it must adapt to the statistics of data to be classified, and the other on the behaviour expected on balanced data. The simplest way to put into practice the former perspective is by adopting accuracy as performance measure. However, in scenarios in which there are practical reasons to focus on type-I or type-II errors (e.g., in biomedicine), other measures may be used instead, including precision-recall diagrams^38^, coverage plots^39^, or generalised $\langle \varphi ,\delta \rangle$ diagrams^32^. On the other hand, an equally simple way to account for the latter perspective is to focus on the accuracy that would be measured as data were in fact balanced. This shift can be implemented by substituting the accuracy with its “unbiased” variant as performance measure. Note that the two kinds of accuracy are strictly related; in particular, with $n$ and $p$ percent of negative and positive samples, the former (say $a_{}$) can be defined as $a_{} = n \cdot \overline{\rho } + p \cdot \rho$, whereas the latter (say $a_{u}$) is obtained by setting $n =p =1/2$ in the previous formula, yielding $a_{u} = \left( \overline{\rho } + \rho \right) /2$. Note that also $\delta$ can be easily reformulated in terms of unbiased accuracy, as $\delta = \rho + \overline{\rho }- 1 = 2 \cdot \left( \rho + \overline{\rho } \right) / 2 - 1 = 2 \cdot a_{u} - 1$, meaning that $\delta$ represents in fact the unbiased accuracy stretched in the interval $[-1,+1]$.

The experimental results reported hereinafter are commented out by following this twofold approach. In particular, Tables 1 and 2 are respectively focused on accuracy and on unbiased accuracy. However, having stressed the strict relation that holds between $a_{u}$ and $\delta$, and considering that this article is centred on $\varphi$ and $\delta$ as performance measures, the table actually reports $\varphi$ and $\delta$ values. Notably, the pair $\varphi$ and $\delta$ can also give useful information about the generalisation capability of a trained MLP. In fact, from a pragmatic point of view, generalisation occurs when no performance reduction is observed while moving the focus from the training to the test set, and at the same time specificity and sensitivity are maximised. Moreover, they should be maximized as much as possible to the same extent, a wanted property being that the classifier should have a null or minimum bias towards the positive or negative category. Equivalently, one may impose that *the sum* of sensitivity and specificity (i.e., $\overline{\rho } + \rho$) should be maximised and *their difference* in absolute value (i.e., $\vert \rho - \overline{\rho } \vert$) minimised. Not incidentally, $\delta = \rho + \overline{\rho }-1$, whereas $\varphi = \rho - \overline{\rho }$. Besides, the above recommendations for assessing the generalisation capability of an MLP, i.e., low φ (absolute value) and high δ﻿, are perfectly compliant with those regarding *success patterns*, the only difference being that for success patterns the sign of δ﻿ is not any longer relevant—as also highly contravariant neurons are very helpful for the classification task. Due to lack of space, on the BP side only the best performing result has been retained for each dataset—choosing between *1HL* and *nHL* architectures (with *1HL* = MLP equipped with one hidden layer, and *nHL* = MLP equipped with more hidden layers). In particular, a better performance of the latter (i.e., *nHL*) has been found only on the datasets autos and bank, for both accuracy and unbiased accuracy. Looking at Table 1, PT appears more effective than BP, as five over ten results are in favour of the former, whereas the others are equivalent. As for Table 2, no clear indication holds to conjecture that one algorithm is better than the other (three datasets are in favour of PT and one in favour of BP). As for the generalisation capability, given the results in terms of $\varphi$ and $\delta$, it is worth pointing out that in some datasets the standard deviations on $\varphi$ reveal a not negligible degree of instability over runs. Summarizing, experimental results show a slight superiority of PT. However, considering that the comparison has been made on a limited number of datasets, and also taking into account that no specific trial-and-error strategy has been put into practice for parameter tuning and MLP shape identification, a cautious conclusion would be that PT is expected to be at least as effective as BP.

**Table 1:** Comparison, with focus on *accuracy*, between BP and PT  applied to non trivial datasets (all from UCI). For each dataset, 100 training and test sets have been generated by random splitting. On the backpropagation side, experiments have been performed on two kinds of MLPs: one equipped with one hidden layer and the other with a shape identical to the one selected for PT. The best results obtained on the backpropagation side has been retained for each dataset. Results in favour of/against PT are highlighted with black/white circles, whereas results with no significant difference are highlighted with an equal sign. Two-sample Welch’s *t*-test has been used to check the similarity between the outcomes of different kinds of classifiers. The significance level for *p*-values has been set to 0.05. Also standard deviation is reported for accuracy. Legenda: *1HL*/*nHL* = MLP with one/more than one hidden layer, $\overline{\rho }$ = specificity, $\rho$ = sensitivity, and $a_{}$ = accuracy.

Dataset	BP (best 1HL/nHL)			PT (nHL)			*p*-value (*a*)	
	$\overline{\rho }$	$\rho$	$a$	$\overline{\rho }$	$\rho$	$a$		
Autos	0.77	0.73	0.76 ± 0.06	0.81	0.68	0.78 ± 0.04	0.0030	•
Bank	0.78	0.74	0.77 ± 0.10	0.76	0.68	0.75 ± 0.07	0.0515	=
Breast-cancer	0.76	0.45	0.67 ± 0.05	0.74	0.51	0.67 ± 0.04	0.2024	=
Census	0.81	0.73	0.75 ± 0.05	0.82	0.75	0.77 ± 0.04	0.0010	•
Connect-4	0.71	0.69	0.70 ± 0.07	0.73	0.71	0.72 ± 0.04	0.0049	•
Credit-approval	0.84	0.85	0.84 ± 0.02	0.85	0.85	0.85 ± 0.02	0.0042	•
Credit-cards	0.73	0.59	0.70 ± 0.18	0.72	0.62	0.70 ± 0.16	0.4837	=
Heart-disease	0.78	0.82	0.80 ± 0.04	0.79	0.82	0.80 ± 0.04	0.5000	=
Sonar	0.83	0.81	0.82 ± 0.05	0.83	0.82	0.83 ± 0.04	0.1426	=
SPECT	0.77	0.71	0.72 ± 0.07	0.75	0.73	0.74 ± 0.04	0.0272	•

**Table 2:** Comparison, with focus on $\delta$ (and on $\varphi$), between BP and PT  applied to non trivial datasets (all from UCI).

Dataset	BP (best 1HL/nHL)				PT (nHL)					
	$\overline{\rho }$	$\rho$	$\varphi$	$\delta$	$\overline{\rho }$	$\rho$	$\varphi$	$\delta$	*p*-value ($\delta$)	
Autos	0.77	0.73	− 0.04 $\pm$ 0.05	0.50 $\pm$ 0.02	0.81	0.68	− 0.13 $\pm$ 0.08	0.49 $\pm$ 0.06	0.0582	=
Bank	0.78	0.74	− 0.04 $\pm$ 0.05	0.52 $\pm$ 0.00	0.76	0.68	− 0.08 $\pm$ 0.19	0.44 $\pm$ 0.06	0.0129	$\circ$
Breast-cancer	0.76	0.45	0.31 $\pm$ 0.16	0.21 $\pm$ 0.11	0.74	0.51	0.24 $\pm$ 0.15	0.25 $\pm$ 0.10	0.0224	•
Census	0.81	0.73	− 0.08 $\pm$ 0.24	0.54 $\pm$ 0.04	0.82	0.75	− 0.06 $\pm$ 0.17	0.57 $\pm$ 0.02	0.0000	•
Connect-4	0.71	0.69	− 0.02 $\pm$ 0.32	0.40 $\pm$ 0.08	0.73	0.71	− 0.02$\pm$ 0.26	0.44 $\pm$ 0.08	0.0001	•
Credit-approval	0.84	0.85	0.01 $\pm$ 0.10	0.69 $\pm$ 0.05	0.85	0.85	− 0.00 $\pm$ 0.06	0.70 $\pm$ 0.05	0.0794	=
Credit-cards	0.73	0.59	− 0.14 $\pm$ 0.44	0.32 $\pm$ 0.11	0.72	0.62	− 0.10 $\pm$ 0.41	0.34 $\pm$ 0.11	0.1032	=
Heart-disease	0.78	0.82	0.04 $\pm$ 0.17	0.60 $\pm$ 0.08	0.79	0.82	0.02 $\pm$ 0.10	0.61 $\pm$ 0.08	0.4008	=
Sonar	0.83	0.81	− 0.02 $\pm$ 0.14	0.64 $\pm$ 0.10	0.83	0.82	− 0.01 $\pm$ 0.11	0.65 $\pm$ 0.09	0.1229	=
SPECT	0.77	0.71	0.06 $\pm$ 0.33	0.48 $\pm$ 0.14	0.75	0.73	− 0.01 $\pm$ 0.15	0.48 $\pm$ 0.11	0.4231	=

The comparison is in fact focused on unbiased accuracy—i.e., on the accuracy measured as datasets were in fact balanced. Results in favour of/against PT are highlighted with black/white circles, whereas results with no significant difference are highlighted with an equal sign. Standard deviation is reported for both $\varphi$ and $\delta$.

#### Experiments on medium-size datasets

To complete the assessment concerning the existence of relevant patterns inside MLPs and the effectiveness of PT, further experiments have been performed on medium-size datasets (all downloaded from the Kaggle ML repository). As made for the experiments performed on UCI datasets, also here two tables have been reported (i.e., Tables 3 and 4)—which account for the behaviour found by focusing on accuracy and on unbiased accuracy (through δ). Given that running experiments on medium-size datasets is a time-consuming task, only 4 runs have been performed for each dataset and algorithm under testing. BP (*nHL* only) and PT have been experimented using the same MLP architecture and the same training parameters. With the exception of a dataset (see below), hidden layers have been equipped with 40, 30, 20, and 10 neurons. As for relevant parameters, learning rate and momentum have been set to 0.01 and 0.05, whereas the number of epochs has been set to 40. Notably, this choice about experimental settings did not affect the emergence of relevant patterns; in fact, for easy problems (e.g., MNIST), the adoption of more layers than needed had the effect of showing a success pattern at the first hidden layer and the same pattern almost unchanged at subsequent layers.

**Figure 9:**
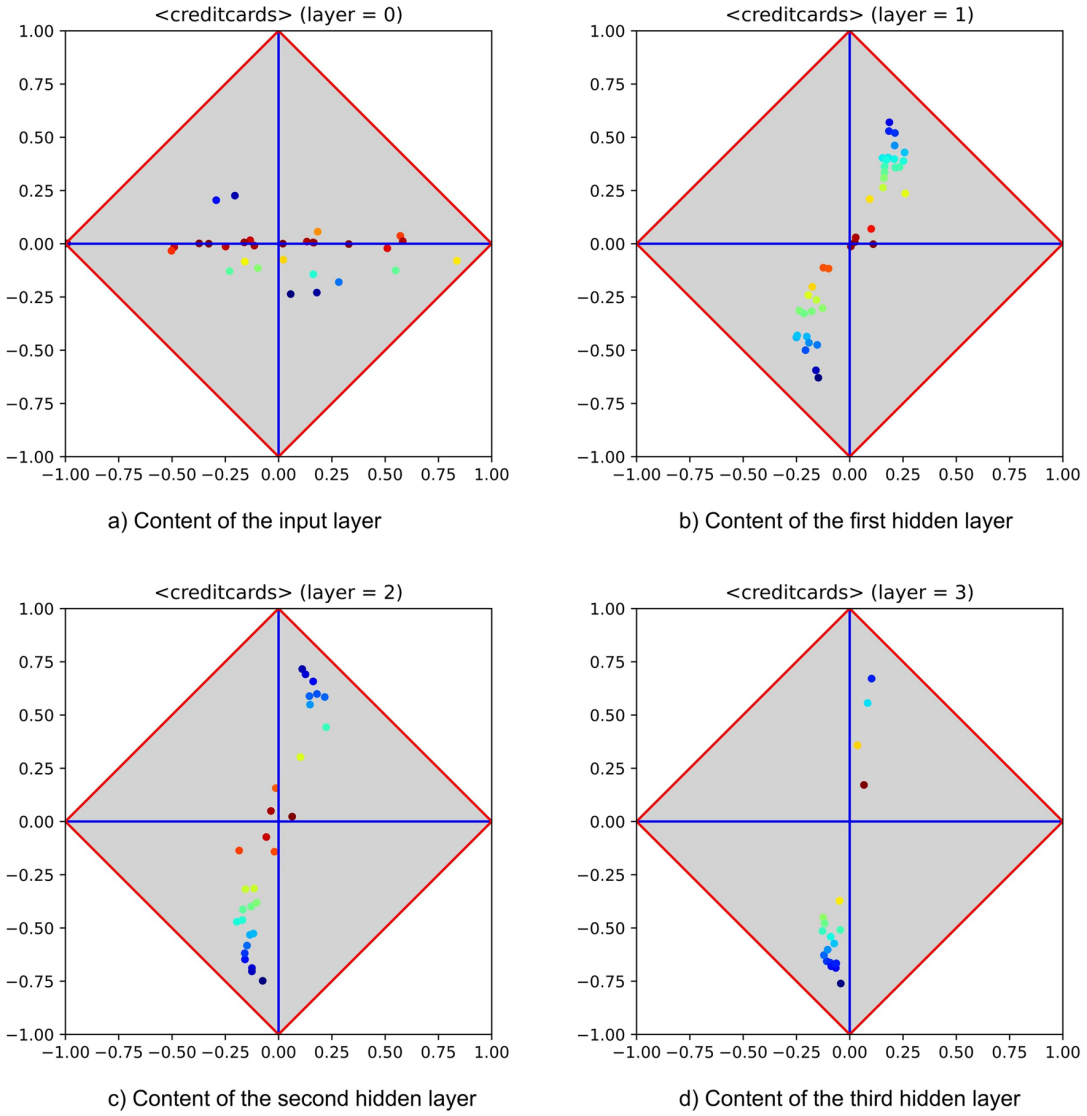
Feature extraction performed by an MLP equipped with four hidden layers (with 40, 30, 20, and 10 neurons) and trained with PT on the Credit Cards Fraud Detection dataset (from the Kaggle ML repository). The sequence highlights that a pattern of success occurs at the first hidden layer and that it is slightly improved at the subsequent layers. The last layer is not reported for the sake of brevity.

**Table 3:** Comparison, with focus on *accuracy*, between BP and PT—applied to medium-size datasets (all from the Kaggle ML repository). The comparison has been performed using the train-and-test strategy, as often done with datasets of significant size. Non binary datasets have been preventively binarized (for the sake of brevity, only the best and the worst cases have been reported for the dataset MNIST). Experimental runs have been repeated 4 times. Also standard deviation is reported for accuracy.

Dataset	#Samples	#Features	BP (nHL)			PT (nHL)		
			$\overline{\rho }$	$\rho$	$a_{}$	$\overline{\rho }$	$\rho$	$a_{}$
MNIST[1] (worst$\vert$BP)	70.000	784	0.96	0.96	$0.96\pm 0.12$	0.98	0.95	$0.98\pm 0.10$
MNIST[2] (best$\vert$both )			0.98	0.96	$0.98\pm 0.08$	0.98	0.96	$0.98\pm 0.10$
MNIST[8] (worst$\vert$PT)			0.98	0.96	$0.98\pm 0.09$	0.95	0.97	$0.95\pm 0.08$
Credit card fraud detection	284.000	30	0.99	0.88	$0.99\pm 0.08$	0.99	0.88	$0.99\pm 0.06$
Heart beat categorization	109.446	187	0.87	0.95	$0.93\pm 0.09$	0.83	0.96	$0.94\pm 0.11$
Diabetic retinopathy[No_DR]	3.662	150.528	0.87	0.88	$0.88\pm 0.13$	0.91	0.88	$0.89\pm 0.13$

Table 3 highlights that the performances of BP and PT are equivalent on the selected datasets, whereas Table 4 points out that high levels of generalisation have occurred on all datasets, being $\delta$ typically high and $\vert \varphi \vert$ always low. As for the occurrence of relevant patterns, this set of experiments has shown no difference between small- and medium-size datasets. As an example, Fig. 9 reports the occurrence of success patterns for the Credit Cards Fraud Detection^40^ dataset. The problem is expected not to be difficult (see the signature of the input layer),  and indeed a success pattern occurs already at the first hidden layer. Notably, notwithstanding the reduction of neurons per layer, the pattern is preserved on subsequent layers (the last hidden layer has not been reported for the sake of brevity). Almost all experiments, except for Diabetic Retinopathy Detection, have been run without performing any feature selection. The cited exception is a collection of images of size $224 \times 224$ and encoded with three RGB values, for a total of 150.528 features. Given such a huge number of features, neither BP nor PT could be able to perform generalisation. Hence, a simple yet effective strategy has been put into practice. First, feature ranking has been performed on the training dataset. Recalling that, for a feature *f*, it is not important to be covariant or contravariant with the positive category, the function $\vert \delta (f) \vert$ has been used to generate the ordering. Then, the first 500 features have been retained and used for training. Also the MLP architecture has been adapted to this dataset. In particular, hidden layers have been equipped with 100, 50, 40, and 20 neurons.

**Table 4:** Comparison, with focus on $\delta$ (and on $\varphi$), between BP and PT applied to medium-size datasets (all from the Kaggle ML repository). The comparison has been performed using the train-and-test strategy. Non binary datasets have been preventively binarized (for the sake of brevity, only the best and the worst cases have been reported for the dataset MNIST). Experimental runs have been repeated 4 times. Also standard deviation is reported for both $\varphi$ and $\delta$.

Dataset	BP (nHL)				PT (nHL)			
	$\overline{\rho }$	$\rho$	$\varphi$	$\delta$	$\overline{\rho }$	$\rho$	$\varphi$	$\delta$
MNIST[1] (worst$\vert$BP)	0.96	0.96	$+0.00\pm 0.05$	0.92 $\pm$ 0.03	0.98	0.96	$0.02\pm 0.05$	0.94 $\pm$ 0.04
MNIST[2] (best$\vert$both )	0.98	0.96	$-0.02\pm 0.03$	0.94 $\pm$ 0.03	0.98	0.96	$-0.02\pm 0.03$	0.94 $\pm$ 0.04
MNIST[8] (worst$\vert$PT)	0.98	0.96	$-0.02\pm 0.02$	0.94 $\pm$ 0.04	0.95	0.97	$+0.02\pm 0.02$	0.92 $\pm$ 0.03
Credit card fraud detection	0.99	0.88	$-0.11\pm 0.02$	0.87 $\pm$ 0.01	0.99	0.88	$-0.11\pm 0.02$	0.87 $\pm$ 0.02
Heart beat categorization	0.87	0.95	$+0.12\pm 0.03$	0.82 $\pm$ 0.01	0.83	0.96	$+0.13\pm 0.02$	0.79 $\pm$ 0.02
Diabetic retinopathy[No_DR]	0.87	0.88	$+0.01\pm 0.07$	0.75 $\pm$ 0.10	0.91	0.88	$-0.03\pm 0.06$	0.79 $\pm$ 0.05

## Discussion

In this section, further comments are given on the following aspects: (i) the capability of MLPs to act as feature encoders when trained with PT; (ii) the occurrence of relevant patterns inside MLPs; (iii) the role of $\langle \varphi ,\delta \rangle$ diagrams in the task of devising more effective training strategies; and (iv) the possibility of using MLPs and $\langle \varphi ,\delta \rangle$ diagrams in combination to perform multivariate analysis.

The insight that lays behind the work described in this article is that MLPs are very effective feature encoders and that relevant patterns arise upon training. The soundness of this insight is also supported by the ongoing research on deep learning, which has fully adopted autoencoders (see Bourlard and Kamp^41^ and Hinton and Zemel^42^) as a mean for implementing feature extraction according to an unsupervised perspective. However, the process of encoding features is not necessarily unsupervised. In fact, applying backpropagation allows to generate different encodings of the input features at each hidden layer, driven by the errors evidenced at the output layer. This process of feature encoding is particularly effective for PT, in which hidden layers are trained one at a time, using mean square error as loss function. In so doing, the transformation of inputs from a layer to another is progressively driven by a generative thrust aimed at making the new encodings more covariant or contravariant with the positive category. Besides, many comments reported in Shwartz-Ziv and Tishby ^21^ have been experimentally assessed while checking the soundness of PT. In fact, on average, the proposed strategy appears as least as effective as BP, allows to better highlight the input compression mechanism, and typically shows a shorter training time.

**Figure 10:**
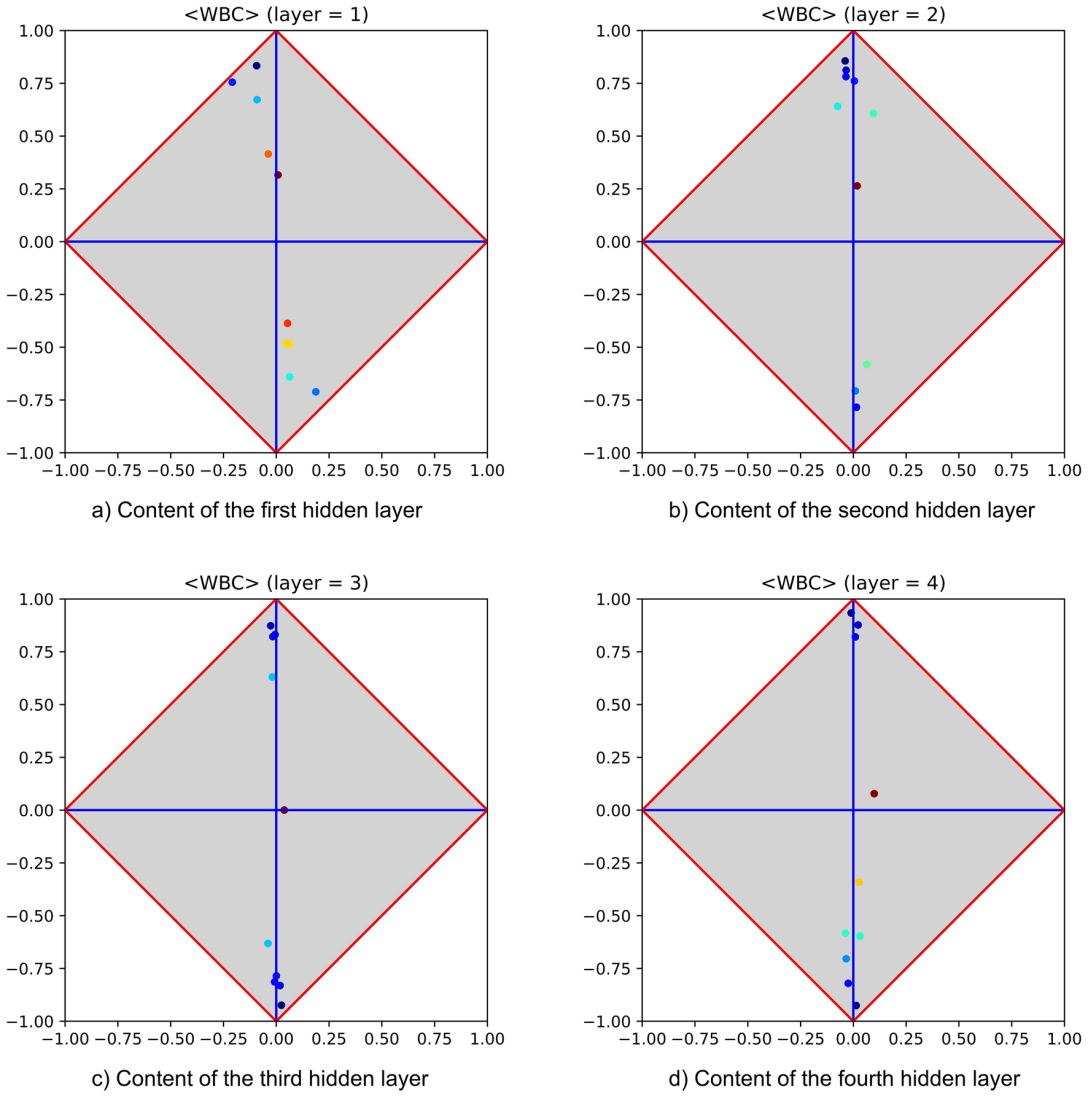
Hidden layers of an MLP trained on the dataset WBC (Wisconsin Breast Cancer) from UCI. To give a flavour of the underlying process, PT has been performed on an MLP architecture equipped with four hidden layers, *all* with the same number of neurons (i.e., 10). The corresponding signatures highlight that the same pattern of generalisation success is duplicated along the hidden layers.

As for the occurrence of relevant patterns inside MLPs, experimental assessments made over a great number of datasets, have shown that the following significant kinds of pattern hold: failure, success and partial success. Failure patterns point to the inability of the MLP to come up with new feature spaces laying as far as possible from the $\varphi$-axis. This kind of patterns has been extensively studied, and the difference between a failure due to the inability of the network to come up with a suitable generalisation has been distinguished from other causes related to algorithmic issues. In particular, the former case is typically evidenced by neurons located close to the centre of the $\langle \varphi ,\delta \rangle$ diagram, whereas the latter (which highlights the occurrence of saturation) is typically evidenced by neurons that lay in proximity of the left and/or right corner(s). Needless to say that these two cases can occur jointly. As for success patterns, they hold when the training activity has generated neurons that are highly covariant or highly contravariant with the positive category. Typically, one cluster is found in proximity of the upper corner and another in proximity of the lower; however, also a single cluster may occur. Experimental results demonstrated that, once achieved, any such pattern tends to be steady. In particular, assuming that a researcher has decided that the MLP at hand should have a shape of length *N* and that a success pattern has been found at a layer $k^{*} < N$, the point here is whether training the remaining layers may improve or not the final performance. Experiments performed on many different problems highlight that negligible improvement can be obtained *after* $k^{*}$, as at all layers $k > k^{*}$ similar patterns of generalisation success would be reproduced. Figure 10 reports the signatures of an MLP equipped with four hidden layers (all with the same number of neurons) and trained on the WBC dataset. The figure shows that only marginal changes characterize a hidden layer with respect to the others, highlighting that a pattern of success holds on all layers. The lack of relevance regarding these variations has been assessed by removing all hidden layers but the first. In fact, as expected, the classification performance was not affected by the removal. Notably, the steadiness of success patterns appears very important for devising adaptive training strategies in which the shape of an MLP is not a priori defined. Patterns of partial success occur when clusters of neurons tend to attain the upper and/or the lower corner, but with limited success (in other words, these clusters typically stand *half-way* between the $\varphi$-axis and the upper and/or lower corners). One reasonable hypothesis that may explain their occurrence consists of assuming that they originate from the intrinsic difficulty of the dataset at hand. A more interesting hypothesis, which however does not rule out the previous one, is that this kind of pattern accounts for the limitations of BP. In the event that at least part of the responsibility for the occurrence of half-way patterns is due to the gradient descent enforced by backpropagation, there is room for experimenting alternative training strategies (e.g., SGD) able to inject the pseudo-random behaviour required to escape from local minima. Also these patterns tend to be steady, thus highlighting that there is no guarantee for PT to come up with a better generalization just adding further layers. Notably, the presence of half-way patterns is consistent with the findings of Salakhutdinov and Murray^43^ and of Larochelle and Bengio^44^, who point out that deep architectures cannot be considered better than shallow ones on every problem.

This proposal also highlights the role of $\langle \varphi ,\delta \rangle$ diagrams in the task of devising more effective training strategies. In particular, the fact that the inner behaviour of MLPs can be investigated with proper visual and computational tools (i.e., $\langle \varphi ,\delta \rangle$ diagrams and measures) promotes the opening of new scenarios, in which further relevant techniques could be borrowed from the machine learning and pattern recognition communities and adapted to this research topic. For instance, assuming that PT is used, one may focus on the advantage of pruning the current layer before training the next. The benefit of applying pruning should not be surprising, as the $\langle \varphi ,\delta \rangle$ analysis performed on hidden layers allowed to verify that they may contain neurons which are apparently not useful for the classification task (e.g., those that lay in proximity of the left or right corner). A simple pruning strategy would consist of devising a proper cost function and use it for ranking neurons with the goal of identifying candidates for deletion. Rather than adopting entropy or Gini index, which appear more convenient for identifying neurons characterised by low accuracy in a MAP setting^45^, one may be interested at minimising the bias as well (i.e., at trying to make specificity and sensitivity as equal as possible while maximising the accuracy). Which cost functions appear more appropriate for enforcing bias minimisation is still a matter of investigation. In any case, the opportunity of visually inspecting hidden layers by means of $\langle \varphi ,\delta \rangle$ diagram should greatly help rapid advances in this research topic. The reader interested in pruning and compression techniques may also consult –for instance– the review articles of Augasta and Kathirvalavakumar^46^ and of Cheng et al.^47^. Alternative pruning strategies may be devised also considering that, at least in principle, any layer of an MLP could be seen as an ensemble—the role of individual classifiers being played by the corresponding neurons. According to this view, an appropriate pruning strategy might be devised in accordance with the proposals concerning the trade-off between diversity and accuracy, as investigated by the community of classifier ensembles (see for example Kuncheva and Whitaker^48^ and Bhatnagar et al.^49^). Notably, this view is indirectly confirmed by the adoption of softmax^50^ in two relevant scenarios: (i) as output blender for classifier ensembles, e.g., Memisevic et al.^51^, and (ii) as output combiner for CNNs, e.g., Krizhevsky et al.^52^ and Liu et al.^53^.

The last comments of this section are devoted to highlight the potential of using MLPs and $\langle \varphi ,\delta \rangle$ diagrams in combination to perform multivariate analysis. In^28^ the author points to the ability of $\langle \varphi ,\delta \rangle$ diagrams to make feature-importance analysis by calculating the class signature on the given dataset (recall that class signatures fall into the broad category of univariate analysis, as they concentrate on each feature taken in isolation). Class signatures are in fact “semi-decidable”, meaning that when at least one feature highly covariant or contravariant with the positive category is found, then the problem at hand is certainly easy. Unfortunately, the converse is not true. In other words, when no good features are found, one may conjecture that the problem is difficult, but further supporting information is needed to complete the assessment. Fortunately, training an MLP on the given dataset can shed more light on its actual difficulty. Again, a failure (i.e., an MLP with limited generalisation capability) would not necessarily mean that the problem is in fact hard to solve. However, the identification of a success pattern at any hidden layer would turn the appraisal from potentially-difficult to easy. This support for decision making is obtained by providing the researcher with manifold class signatures, one for each layer (including the one evaluated on inputs.) Although each signature is made according to a univariate perspective, those evaluated at the hidden layers can give strong support to multivariate analyses, as the neurons therein may provide useful combinations of input features (depending on the work done by the adopted training algorithm). An evidence about the shortcomings of univariate analysis has been given before with the synthetic *xor* problem. This is a characteristic example of how univariate analysis may fail while multivariate analysis (obtained by the combined use of MLPs and $\langle \varphi ,\delta \rangle$ diagrams) may succeed.

## Methods

This section is devoted to give some details about PT, whose most distinctive characteristics is the ability of performing greedy layer-wise training.

**Figure 11:**
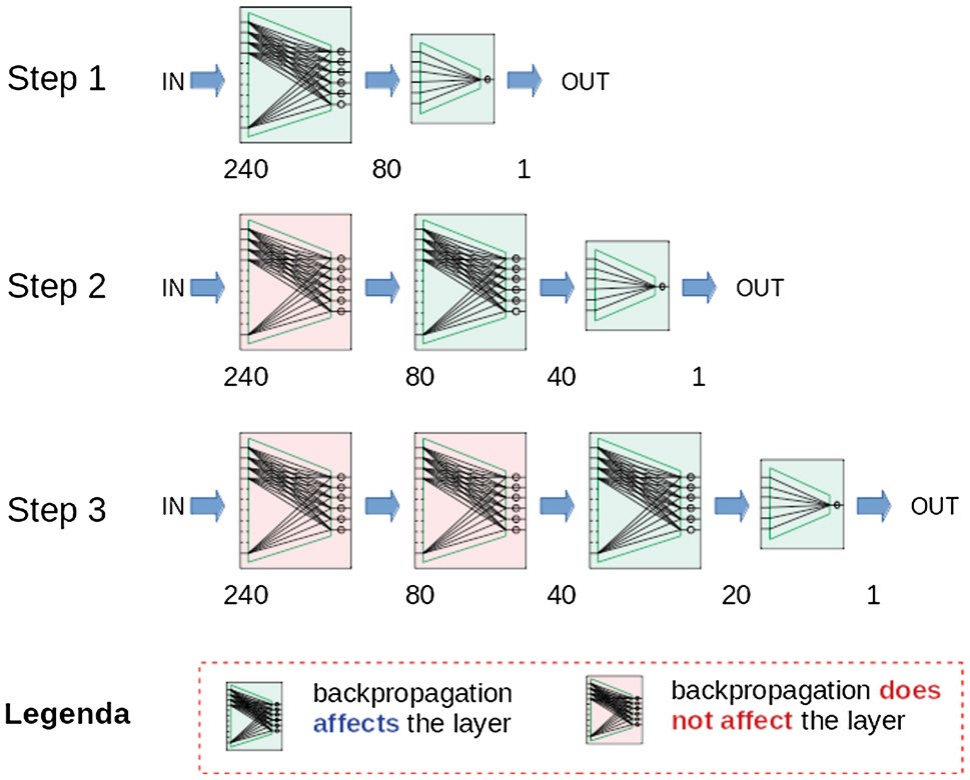
Snapshot of PT applied to an MLP equipped with three hidden layers (240 inputs, one output, and three hidden layers with 80, 40, and 20 neurons).

To understand PT from an algorithmic perspective, let us concentrate on Fig. 11, which gives a snapshot of the training activity under the hypothesis that the shape of the MLP at hand is $\langle 240, 80, 40, 20, 1 \rangle$—meaning 240 inputs, first hidden layer with $\langle 240 \times 80 \rangle$ weights, second with $\langle 80 \times 40 \rangle$ weights, third with $\langle 40 \times 20 \rangle$ weights, and output layer with $\langle 20 \times 1 \rangle$ weights. The process starts by training the first layer. Then this layer is frozen and used as feature encoder to provide the inputs for training the second layer. Again, this second layer is frozen and used (in pipeline with the first) as feature encoder to provide the inputs for training the third layer, and so on until the output layer is reached. Notably, PT is compliant with the vision of Shwartz-Ziv and Tishby^21^, who adopt a “supervised” perspective while analysing the content of a multilayer neural network from an information theoretic perspective. In particular, assuming that *X* and *Y* denote input and output of a multi layer ANN, the authors point out that any internal representation of the input, say *T*, can be seen as the combination of an encoder, $P(T \vert X)$, and a decoder $P( \tilde{Y} \vert T)$, with $\tilde{Y}$ representing the output of the network. In fact, PT provides an effective greedy strategy aimed at generating the encoder entrusted with feeding the final layer. To this end, at each iteration, an MLP with *one* hidden layer (say *trainee*) is trained, whose input is taken from the encoder generated so far (of course, at the first iteration, the actual input is given to the trainee). At the end of the iteration, the hidden layer of the trainee is appended to the encoder, whereas its decoder is neglected. Then, the training algorithm starts over again with the next iteration, until the final architecture has been generated. Note that, at the last iteration, the output layer (i.e., the decoder) of the trainee is appended to the encoder. In so doing, PT gives an effective solution to the problem of finding useful representations of the given input according to the information provided by sample labelling. This conceptualization has been implemented defining a Python class, say MLPP, in which several trainees (i.e., different MLPs equipped with a single hidden layer) are trained, and their hidden layer progressively embedded into a proper internal slot (of the MLPP object) that provisionally plays the role of input encoder—see also the Supplementary Information (Supplementary section S3) available online. It is worth pointing out that, unlike other proposals that typically rely on autoencoders, here feature extraction is performed according to a supervised perspective. In fact, in PT the information about class labelling is made part of the whole process at each iteration.

As for its roots, PT shares the practice of performing layer-wise training with several proposals. A pioneering work on this topic has been made by Fahlman and Lebiere^54^ who proposed the cascade correlation (CC) architecture and algorithm. Instead of just adjusting the weights in a network of fixed topology, the CC algorithm begins with a minimal network, and then automatically trains and adds new hidden units one by one, creating a multi-layer structure. Once a new hidden unit has been added to the network, its input-side weights are frozen, so that the unit becomes a permanent feature detector in the network under construction. In 2006, Hinton et al.^55^ propose a layer-wise learning algorithm for training deep belief networks. The authors illustrate a fast and greedy learning algorithm for constructing multilayer directed networks, one layer at a time. This algorithm is used to initialize a slower learning procedure aimed at performing fine-tuning. Bengio et al.^30^ propose to pre-train DNNs using a layer-wise unsupervised strategy, followed by fine tuning. In a 2010 article, Erhan et al.^56^ analyse the motivations why pre-training helps the learning process. Following the insight on pre-training, Furusho et al.^57^ investigate how relevant information theoretic measures are related to the generalization error, how the representations change as the number of hidden layers increases, and how pre-training affects input encoding. Arnold and Ollivier^58^ propose a layer-wise training procedure based on the best latent marginal, which is able to approximate the global optimum. Duan et al.^59^ propose a novel family of connectionist models based on kernel machines and consider the problem of learning concepts layer by layer. In particular, the authors propose a method to “kernelize” any ANN, which allows to obtain a counterpart of any given ANN that is actually powered by kernel machines instead of neurons. Considering the two-layer case without loss of generality, the authors illustrate a framework (and an algorithm) able to minimise the objective function of the network according to a greedy training scheme that learns one layer at a time.

As final comments, note that the vanishing (or exploding) gradient problem (see for example Hochreiter et al.^60^) is strongly contrasted by PT, as each layer is trained separately from the others. Hence, the push for change that motivated the adoption of ReLU in place of a sigmoid or a hyperbolic function as “right” activation function should become a minor issue under this framework. Moreover, using PT, the adoption of proper pruning strategies is expected to make void the problem of residual neurons found to be in saturation.

## Conclusion

This article has shown that relevant patterns arise inside MLPs upon training, as clearly highlighted by the $\langle \varphi ,\delta \rangle$ signatures of their hidden layers. In particular, the analysis has experimentally demonstrated that, upon training, clear patterns representing success or failure typically hold. The existence of patterns that lay half-way between success and failure has also been highlighted. To facilitate the analysis on MLPs equipped with more than one hidden layer, a layer-wise training strategy has been devised and implemented, called progressive training. In this strategy, layers are individually trained starting from the one in charge of processing the given inputs. Despite the fact that this is a methodological article, experimental results show that progressive training appears a viable alternative to the backpropagation algorithm. As for future work, several research activities are under way, including (i) getting a better understanding of the connection between MLP relevant patterns and training parameters, (ii) devising a general criterion for stopping the training as soon as a success pattern is found, (iii) devising proper pruning strategies, entrusted with the deletion of neurons deemed useless; (iv) investigating the causes that generate half-way patterns; and (v) characterising progressive training from an information theory perspective. Notably, after reaching theoretical and/or experimental findings on the cited issues, setting up an adaptive training strategy in which the final shape of a neural architecture is not known in advance should no longer be an unattainable goal.

## Supplementary information

Supplementary Information.

## Data Availability

The data that support the findings of this study are available from the following Machine Learning Repositories: (a) UC Irvine (URL https://archive.ics.uci.edu/ml/datasets.php) and (b) Kaggle (URL https://www.kaggle.com/data). All datasets used in this research are publicly available.
