# Supplementary material for: Using phidelta diagrams to discover relevant patterns in multilayer perceptrons
Source: Sci Rep. 2020 Dec 7;10:21334. doi: 10.1038/s41598-020-76517-0 (PMC7721750; doi:10.1038/s41598-020-76517-0)
Supplement: Supplementary file 1 — Supplementary Information. [file 41598_2020_76517_MOESM1_ESM.pdf]

Supplementary material for the article:

Using phidelta diagrams to discover relevant patterns in multilayer perceptrons

by **Giuliano Armano**

Dept. of Mathematics and Computer Science, University of Cagliari, Cagliari, Italy (e-mail: armano@unica.it)

S1 Some details on $\langle \varphi, \delta \rangle$ measures and diagrams

This section contains some details about $\langle \varphi, \delta \rangle$ diagrams. Additional information can be found in published articles on $\langle \varphi, \delta \rangle$ diagrams.

Devised as a variant of ROC curves, $\langle \varphi, \delta \rangle$ diagrams are a diamond-shaped 2D tool used for classifier performance evaluation and for feature assessment. Figure 1a reports the semantics of relevant points under the former perspective. In particular,

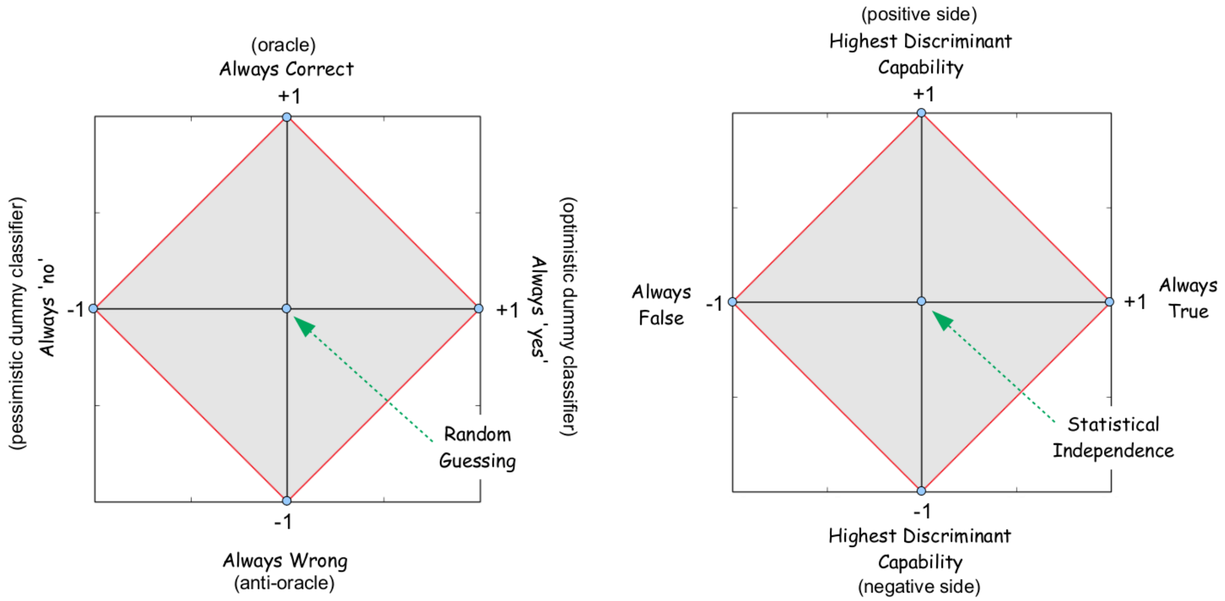

Figure 1. Relevant points in the $\langle \varphi, \delta \rangle$ space, from a classifier and from a feature-oriented view (left-and and right-hand side, respectively).

a classifier whose performance in terms of φ and δ lays in proximity of the upper / lower corner in fact approximates the behaviour of an oracle / anti-oracle (i.e., any such classifier would almost never / always fail). Conversely, a performance reported in proximity of the left / right corner characterises "dummy" classifiers (i.e., any such classifier would almost always answer $No = -1$ / $Yes = +1$, regardless of the sample under analysis). As for the centre of the diagram, it corresponds to a classifier that performs random guessing. Figure 1b reports the semantics of relevant points under the latter perspective. In fact, it strictly depends on the former, as each feature can be seen as a single-feature classifier. In particular, a feature reported in proximity of the upper / lower corner is strongly covariant / contravariant with the positive category. Any such feature would have high discriminant capability, regardless of the sign of δ. Conversely, a feature reported in proximity of the left- / right-hand corner would typically be irrelevant for the classification task, as its value is almost always -1 / $+1$ in the dataset at hand, regardless of the class label of samples. Moreover, features that are statistically independent of the positive (and negative) category would be located (close to) the centre of the diagram.

For the sake of completeness, let us recall the definitions of φ and δ, given in terms of specificity ($\overline{\rho}$) and sensitivity (ρ):

$$\begin{cases} \varphi & = \rho - \overline{\rho} \\ \delta & = \rho + \overline{\rho} - 1 \end{cases} \tag{1}$$

Let us preliminary note that the characteristic diamond shape of $\langle \varphi, \delta \rangle$ diagrams depends on the constraints that apply to φ and δ through specificity and sensitivity. For instance, considering the right-upper side of the diagram (in which $0 \leq \varphi \leq 1$ and $0 \leq \delta \leq 1$), one can write:

$$\varphi + \delta = \rho - \overline{\rho} + \rho + \overline{\rho} - 1 = 2\,\rho - 1 \tag{2}$$

The range of variation for ρ in this side of the diagram is $[0.5, 1.0]$. Hence, for the right-upper side $0 \leq \varphi + \delta \leq 1$, while in general $|\varphi| + |\delta| \leq 1$, which corresponds to the constraints that give rise to the characteristic diamond shape.

Interpretation of φ and δ for classifier performance evaluation

Reporting the performance of a classifier or feature with respect to the positive category as a point in a $\langle \varphi, \delta \rangle$ diagram gives relevant information to a researcher. In particular, φ and δ basically correspond to bias and accuracy estimates, obtained as if negative and positive samples were perfectly balanced. In fact, Equation (1) highlights that φ and δ are *not* affected by the imbalance between negative and positive samples, whereas the standard definitions of bias and accuracy are. However, alternative definitions for these performance measures can be obtained from the classical ones by forcing perfect balancing. Without claiming to be complete, let us give a short trace aimed at formally verifying the assertions made for φ and δ.

Correspondence between φ *and* bias

Being $\hat{f}$ and f a classifier and the corresponding oracle, a sound definition for bias can be given in terms of expected value as follows:

$$bias[\hat{f}] = E[\hat{f}(\mathbf{X}) - f(\mathbf{X})] = E[\hat{f}(\mathbf{X})] - E[f(\mathbf{X})] \tag{3}$$

where $\mathbf{X}$ is a random variable whose values are single dataset realisations. Beyond this general definition, one may also try to estimate the bias of a classifier over a specific dataset (the more the dataset is statistically significant, the more the estimate is reliable). Be n and p the percent of negative and positive samples of this dataset. Moreover, tn, fn, fp and tp denote the true / false negative rate and the false / true positive rate evaluated from the confusion matrix that summarizes the outcomes of the experiment. Assuming that a negative sample counts -1 whereas a positive sample counts $+1$, we can write:

$$bias\left[\hat{f}\right] \approx \left[\ (p-n) + 2\cdot n\cdot fp - 2\cdot p\cdot fn\ \right] - (p-n) = 2\cdot n\cdot fp - 2\cdot p\cdot fn \tag{4}$$

Being interested to the version of the measure evaluated in absence of imbalance (say $bias_u$), let us derive it from the definition above by imposing that $n = p = 1/2$:

$$bias_u\left[\hat{f}\right] \approx 2\cdot\frac{1}{2}\cdot fp - 2\cdot\frac{1}{2}\cdot fn = fp - fn = (1-tn) - (1-tp) = tp - tn \tag{5}$$

As tn and tp are in fact specificity and sensitivity, we can write:

$$bias_u\left[\hat{f}\right] \approx tp - tn = \rho - \overline{\rho} \equiv \varphi \tag{6}$$

Equation (5) highlights that φ actually estimates the bias of a classifier with respect to the oracle, on a given dataset and in conditions of perfect balancing. As a consequence, when the performance of a classifier measured over a test set lies on the positive semiplane of φ, one can argue that it has a bias towards the positive category and vice versa.

Correspondence between δ *and* accuracy

Under the same assumptions made for calculating the bias over a specific dataset, let us first write down the classical formula of accuracy in terms of specificity and sensitivity:

$$a = \frac{TN + TP}{NEG + POS} \equiv n\cdot tn + p\cdot tp = n\cdot\overline{\rho} + p\cdot\rho \tag{7}$$

where NEG and POS denote the number of negative and positive samples, whereas TN and TP denote the number of true negatives and true positives.

Again, the accuracy in conditions of perfect balancing (say a_u) can be obtained by imposing that $n = p = 1/2$. In symbols:

$$a_u = \frac{1}{2}\cdot\overline{\rho} + \frac{1}{2}\cdot\rho = \frac{\overline{\rho}+\rho}{2} \tag{8}$$

Finally, the definition of δ can be obtained by linearly stretching a_u in the interval $[-1,+1]$, as follows:

$$2\cdot a_u - 1 = 2\cdot\frac{\overline{\rho}+\rho}{2} - 1 = \overline{\rho} + \rho - 1 \equiv \delta \tag{9}$$

As a consequence, when the performance of a classifier measured over a test set lies on the positive semiplane of δ, its performance is better than random guessing and vice versa.

Interpretation of φ and δ for feature assessment

The same semantics can be retained while performing feature assessment. In this case, given a training set, $\langle \varphi, \delta \rangle$ diagrams allow to depict the corresponding "class signature", obtained by reporting the behaviour of each feature, seen as an elementary classifier, with respect to the positive category. According to this view, the horizontal axis of a $\langle \varphi, \delta \rangle$ diagram (i.e., φ) is the locus of points such that the mutual information between a feature and the positive or negative category drops to zero. In particular, features that lay close to the left-hand side of a $\langle \varphi, \delta \rangle$ diagram have most often a value of -1, regardless of the labelling of samples. Conversely, features that lay close to the right-hand side of a $\langle \varphi, \delta \rangle$ diagram have most often a value of $+1$, regardless of the labelling of samples. As for features that lay close to the centre of the diagram, they are in fact statistically independent of the positive or negative category. For the above reasons, features laying close to this axis, i.e., with small $|\delta|$, are expected to give limited or negligible support to the classification process. As for the vertical axis (i.e., δ), it illustrates to what extent a feature is covariant or contravariant with the positive category. In particular, features whose δ value is close to the upper or lower corner (i.e., with high $|\delta|$) are expected to give strong support to the classification process. It is worth pointing out that the feature importance estimates shown by the class signature can be useful only in a setting of univariate analysis, as they are obtained considering each feature in isolation. To perform multivariate analysis, $\langle \varphi, \delta \rangle$ diagrams should be used in combination with MLP architectures –the latter being the most established tool available off the shelf for identifying relevant combinations of features, thanks to their ability of acting as feature encoders.

S2 Some details on the command files used to run experiments in batch mode

This section contains a brief description of the command files used to run experiments in batch mode. An excerpt of a command file is also given.

Performing multiple experiments, each with several runs, and collecting the corresponding results to facilitate their analysis is a viable task only if the number of datasets is very low. In any other case, batch command files are adopted as typical practice.

Listing 1. Excerpt of the batch command file used to perform comparative experiments

```
@dataset 'autos'

  --repeat(10)
  --do(mode='kfold', shape=(60,1), strategy='backprop')
  --netargs(learning_rate=0.01, momentum=0.05, num_epochs=100)

  --repeat(10)
  --do(mode='kfold', shape=(50,30,10,1), strategy='backprop')
  --netargs(learning_rate=0.01, momentum=0.05, num_epochs=100)

  --repeat(10)
  --do(mode='kfold', shape=(50,30,10,1), strategy='progressive')
  --netargs(learning_rate=0.01, momentum=0.05, num_epochs=40)

@dataset 'breast-cancer'

  --repeat(10)
  --do(mode='kfold', shape=(80,1), strategy='backprop')
  --netargs(learning_rate=0.01, momentum=0.05, num_epochs=100)

  --repeat(10)
  --do(mode='kfold', shape=(60,40,20,1), strategy='backprop')
  --netargs(learning_rate=0.01, momentum=0.05, num_epochs=100)

  --repeat(10)
  --do(mode='kfold', shape=(60,40,20,1), strategy='progressive')
  --netargs(learning_rate=0.01, momentum=0.05, num_epochs=40)
```

The format of batch command files used in this research to run experiments is very simple, yet enough powerful to allow to specify all relevant information. Listing 1 reports an excerpt of a specific batch command file. Although the corresponding meaning is straightforward, let us spend a few words on it:

- A batch command file is made by a sequence of *dataset commands*. Each dataset command starts with "@dataset" followed by a dataset name, which is a unique identifier used to load the corresponding data.
- One or more *run commands* can be embodied within the scope of the specified dataset.
- A run command consists of i) number of runs to be performed, ii) information about training mode, MLP architecture and training strategy, together with iii) relevant network parameters.
- As for the number of runs, they must be given according to the syntax: `--repeat(num-of-runs)`.
- As for the training mode, its values can be either 'train', 'test', or 'kfold'. Note that the 'train' mode is used to test the performance of an MLP on the whole dataset (in other words, in this mode training set and test set coincide). Hence, this mode should be used for preliminary experiments only.
- As for the shape, the number of neurons must be specified for all layers but the input one. In fact, the actual number of input features is retrieved by the dataset loader.
- The strategy to be followed can be either 'backprop' or 'progressive' (no more strategies are available, so far).
- The typical MLP parameters can also be specified –namely, 'learning rate' (LRate), 'momentum' (momentum), and 'number of epochs' (num_epochs). Their default values are 0.01, 0.05 and 100, respectively.

Further information can be given in the line that starts a dataset command. In particular, a specific command can be temporarily disabled, which is useful to avoid any unwanted proliferation of batch command files (syntax: `--skip`).

S3 Some details about the implementation of the progressive training strategy

In this section, some technical details about the implementation of the progressive training strategy are given. To facilitate readers that are not familiar with the Python language, the reported source code has been greatly simplified.

Progressive training is a greedy layer-wise training strategy devised to facilitate the behaviour of MLPs as feature encoders. This strategy trains each layer in isolation, proceeding from the input towards the output layer.

Be *net* the whole network to be trained, equipped with $n > 2$ hidden layers. The training process consists of training n MLPs (say *trainees*), each equipped with a single hidden layer, following the direction that goes from the actual inputs to the desired output. At the first iteration, the corresponding trainee sees the actual inputs and is trained according to the given sample labelling. The resulting hidden layer is saved in a net slot called *encoder*. The second step mimics the first, with the significant difference that now the input of the trainee is obtained by encoding the actual inputs by means of the current net encoder. At the end of the second step, the hidden layer of the trainee is appended to the net encoder, thus forming a two-layer encoder. The process is repeated identically for each subsequent layer to be trained, until the last layer is reached. Upon completion of the last training step, the final network is obtained by appending to the encoder the output layer of the last trainee. Notably, each trainee is currently trained with classical backpropagation; however, nothing prevents from using alternative training strategies.

Listing 2. MLP trained with a progressive strategy (simplified listing in Python)

```
from neuralnets.mlp import MLP

class MLPP(object):

  "Multilayer perceptron trained with a progressive strategy"

  def __init__(self, *shape, LRate=0.01, momentum=0.05, num_epochs=100):
    "Initialize the MLPP with the given shape and network parameters"
    self.shape = shape
    self.netargs = { 'LRate' : LRate, 'momentum' : momentum, 'num_epochs' : num_epochs }
    self.encoder, self.layers = list(), list()

  def train(self, data, labels):
    "Train the MLPP according to a progressive strategy"
    tdata, tlabels = data, labels # start the first training with actual inputs
    for k in range(len(self.shape[:-1])): # iterate over all hidden layers
      tshape = self.MLP_shape(at=k) # get the shape of the trainee at the k-th step
      trainee = MLP(*tshape, **self.netargs) # create the trainee with proper shape and network args
      trainee.train(tdata,tlabels) # train the trainee with proper data and labels
      self.encoder += [ trainee.hidden_layer ] # append the trainee hidden layer to the embedded encoder
      tdata = self.transduce(data,encoder=self.encoder) # generate new data using the embedded encoder
    self.layers = self.encoder + [ trainee.output_layer ] # self.layers is in fact the trained MLPP ...
```

As an example of progressive training, let us suppose that the network to be trained has the following shape: $\langle 240, 80, 40, 20, 1\rangle$ –meaning that an input layer of 240 neurons is followed by a first hidden layer of 80 neurons, a second of 40 neurons and a third of 20 neurons. According to the given algorithm, three trainees must be trained: the first with shape $\langle 240, 80, 1\rangle$, the second with shape $\langle 80, 40, 1\rangle$, and the third with shape $\langle 40, 20, 1\rangle$. Listing 2 reports a simplified version of the training algorithm, written in Python. The corresponding Python class is called MLPP (standing for MLP progressive). The internal slot devoted to progressively store the encoder under construction is denoted as `self.encoder` and the k-th trainee is denoted as `trainee`. Note that the final network, denoted as `self.layers`, has been differentiated from the encoder, to point out that also the output layer of the last trainee has been made part of it.

As a final note, let us point out that all experiments with progressive training can be reproduced after installing the software provided at GitHub (URL: https://github.com/garmano/neuralnets). A new release of the libraries reported therein, implemented using Keras (https://keras.io) and Pandas (https://pandas.pydata.org/), is under way.
